# Use of Placebo and Nonoperative Control Groups in Surgical Trials: A Systematic Review and Meta-analysis

**DOI:** 10.1001/jamanetworkopen.2022.23903

**Published:** 2022-07-27

**Authors:** Teemu Karjalainen, Juuso Heikkinen, Ljoudmila Busija, Jarkko Jokihaara, Adriane M. Lewin, Justine M. Naylor, Laura Harris, Ian A. Harris, Rachelle Buchbinder, Sam Adie

**Affiliations:** 1 Department of Surgery, Hospital Nova Central Finland, Jyvaskyla, Finland; 2 Monash Department of Clinical Epidemiology, Cabrini Institute, Malvern, Australia; 3 School of Public Health and Preventive Medicine, Department of Epidemiology and Preventive Medicine, Monash University, Malvern, Australia; 4 Department of Orthopaedics, Oulu University Hospital, Oulu, Finland; 5 Biostatistical Consulting Platform, School of Public Health and Preventive Medicine, Monash University, Melbourne, Australia; 6 Tampere University and Tampere University Hospital, Tampere, Finland; 7 Ingham Institute for Applied Medical Research, South Western Sydney Clinical School, UNSW Sydney, Liverpool, New South Wales, Australia; 8 South Western Sydney Clinical School, UNSW, Liverpool Hospital, New South Wales, Australia; 9 Sydney Orthopaedic Trauma and Reconstructive Surgery, Kogarah, New South Wales, Australia; 10 Ingham Institute for Applied Medical Research, South Western Sydney Clinical School, UNSW Sydney, Liverpool, New South Wales, Australia; 11 Department of Epidemiology and Preventive Medicine, School of Public Health and Preventive Medicine, Monash University; 12 Monash Department of Musculoskeletal Health and Clinical Epidemiology, Cabrini Health, Melbourne, Australia; 13 St George and Sutherland Clinical School, UNSW Sydney, Kogarah, New South Wales, Australia

## Abstract

**Importance:**

Nonspecific effects, particularly placebo effects, are thought to contribute significantly to the observed effect in surgical trials.

**Objective:**

To estimate the proportion of the observed effect of surgical treatment that is due to nonspecific effects (including the placebo effect).

**Data Sources:**

Published Cochrane reviews and updated, extended search of MEDLINE, Embase, and CENTRAL until March 2019.

**Study Selection:**

Published randomized placebo-controlled surgical trials and trials comparing the effect of the same surgical interventions with nonoperative controls (ie, no treatment, usual care, or exercise program).

**Data Extraction and Synthesis:**

Pairs of authors independently screened the search results, assessed full texts to identify eligible studies and the risk of bias of included studies, and extracted data. The proportion of all nonspecific effects was calculated as the change in the placebo control divided by the change in the active surgery and pooled in a random-effect meta-analysis. To estimate the magnitude of the placebo effect, we pooled the difference in outcome between placebo and nonoperative controls and used metaregression to estimate the association between the type of control group and the treatment effect (difference between the groups), adjusting for risk of bias, sample size, and type of outcome.

**Main Outcomes and Measures:**

Between- and within-group effect sizes expressed as Hedges *g*.

**Results:**

In this review, 100 trials were included comprising data from 62 trials with placebo controls (3 also included nonoperative controls), and 38 trials with nonoperative controls (32 interventions; 10 699 participants). Risk of bias across trials was comparable except for performance and detection bias, which was high in trials with nonoperative controls. The mean nonspecific effects accounted for 67% (95% CI, 61% to 73%) of the observed change after surgery; however, this varied widely between different procedures. The estimated surgical placebo effect had a standardized mean difference (SMD) of 0.13 (95% CI, −0.26 to 0.51). Trials with placebo and nonoperative controls found comparable treatment effects (SMD, −0.09 [95% CI, −0.35 to 0.18]; 15 interventions; 73 between-group effects; adjusted analysis: SMD, −0.11 [95% CI, −0.37 to 0.15]).

**Conclusions and Relevance:**

In this review, the change in health state after surgery was composed largely of nonspecific effects, but no evidence supported a large placebo effect. Placebo-controlled surgical trials may be redundant when trials with nonoperative controls consistently report no substantial association from surgery compared with nonoperative treatment.

## Key Points

**Question:**

How large are nonspecific effects, including placebo effects, in surgical randomized trials?

**Findings:**

In this meta-analysis of 100 trials, those with nonoperative and nonblinded controls were found to yield results largely comparable with placebo-controlled trials. Nonspecific effects constituted a mean of two-thirds of the total effect of surgery.

**Meaning:**

This study found that improvements not caused by the surgery itself were mostly nonspecific effects other than placebo effects, suggesting that when surgical trials with nonoperative controls demonstrate no benefits, placebo-controlled studies may be redundant.

## Teaser

This systematic review and meta-analysis estimates the proportion of the observed effect of surgical treatment due to nonspecific effects, including the placebo effect.

## Introduction

Health changes after surgery comprise 2 parts—specific and nonspecific effects. Specific effects are a direct result of the physical changes caused by the key therapeutic element of the surgical procedure (eg, repair of a torn tendon). Nonspecific effects are not dependent on the key therapeutic step and can further be divided into placebo effects and other nonspecific effects, such as regression to the mean and the natural history of the disease.^1,2^

**Figure 1.**
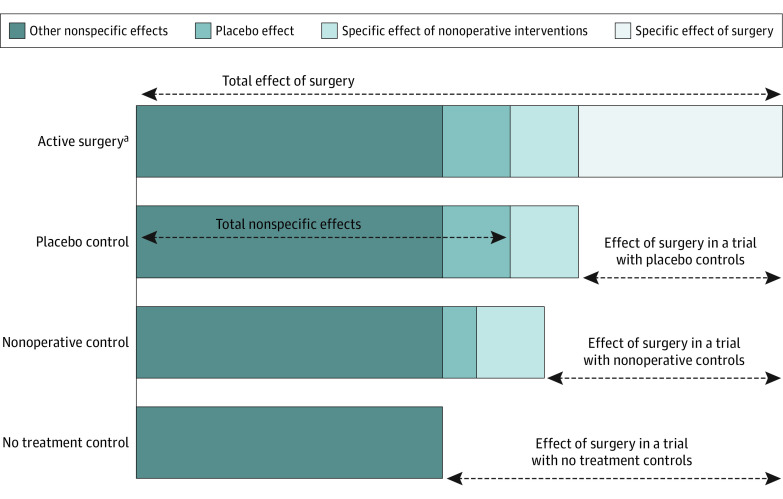
Observed Effects in a Hypothetical Study Other nonspecific effects include (but are not restricted to) the natural course of the disease, regression to the mean and placebo effects of other nonoperative interventions. The estimated effect of surgery depends on the type of control group: nonplacebo controls yield a larger surgical effect if the surgical placebo effect is large (present only in the group receiving surgery). ^a^Assumes that surgical group also receives nonoperative regimen.

To offset various nonspecific effects, trialists need to use a control group to measure the causal effect of surgery (ie, treatment effect). While the use of nonblinded and nonoperative controls enables the estimation of the effect of undergoing surgery compared with nonoperative treatment, use of a placebo control identifies the fundamental efficacy of the surgical intervention by accounting for all nonspecific effects, including the surgical placebo effect (Figure 1).

Rigorous placebo-controlled studies increase the certainty about the true therapeutic effect or efficacy of surgery, thereby providing best evidence for decision-making in clinical practice.^3^ It is not always possible or necessary (eg, for objective outcomes, such as death) to assess the effect of surgery with a placebo control. However, when surgery is used to improve pain and quality of life for non–life-threatening or self-limiting conditions, nonblinded trials could overestimate the benefits of surgery. The use of placebo controls has been considered particularly important in these cases. Surgery may elicit a pronounced placebo effect because of the imposing therapeutic envelope of inherent risk, the technical aspects of the intervention, and the higher costs compared with nonoperative treatment.^4,5,6^ However, there is no systematic evidence to support this hypothesis—previous studies suggesting a large placebo effect measured improvement after receiving placebo intervention that includes both placebo and other nonspecific effects combined.^7,8,9,10,11,12^ The aims of this study were to estimate (1) the mean proportion of the observed effect of surgery that can be attributed to total nonspecific factors; (2) the magnitude of the surgical placebo effect; and (3) the difference in the treatment effect between placebo- and nonoperative-controlled surgical trials testing the same surgical intervention.

## Methods

### Study Design and Definitions Used in This Study

We designed a meta-epidemiological study that included meta-analysis and metaregression. This systematic review and meta-analysis followed the Preferred Reporting Items for Systematic Reviews and Meta-analyses (PRISMA) reporting guideline where applicable.^13^ Key concepts are detailed in Table 1.

**Table 1.** Definitions Used in This Study

Term	Definition
Effect of placebo surgery	The observed effect in participants who receive a placebo intervention. This is the total sum of all nonspecific effects (ie, placebo effect and other nonspecific effects).
Effect of surgery	The difference between active surgery and control group in a trial, ie, the quantitative measure of the effect that surgery has on an outcome measure. The magnitude can vary based on the type of control group.
Placebo effect	The effect experienced because psychological processes (based on expectations, conditioning or patient-caregiver interaction) related to receiving an intervention.
Other nonspecific effects	These include (but are not restricted to) the natural course of the disease, regression to the mean and the effect of other interventions (which includes effects and placebo effects of nonoperative cointerventions). This is the observed effect in the nonoperative control group.
Placebo surgery	A placebo surgical procedure that follows all the steps of a normal procedure, except (at minimum) the key therapeutic step is omitted. The fidelity of placebo surgery (extent of similarity to the active intervention) may vary from skin incision only (low fidelity) to including all other steps of the procedure except the presumed active component (high fidelity).^8^
Specific effect of surgery	The effect provided by the key therapeutic step of a surgical procedure. This is the difference between active surgery and placebo control group.
Surgery	Any intervention that changes anatomy (including use of an implant to achieve that) or requires a skin or mucosal (epithelial) incision or suturing to achieve its effect.
Total effect of surgery	The sum of all effects, nonspecific and specific. This is the observed effect in the active surgery group.

### Inclusion Criteria and Identification of Eligible Studies

We included randomized clinical trials that assessed the efficacy of any surgical intervention by including a placebo control. We also included comparative effectiveness trials where the same surgical intervention (as in the identified placebo trials) was compared with either no treatment, usual care, or exercise programs. Trials where the nonoperative control received cointerventions not provided to the surgical group were excluded because these could not estimate the effect of having surgery.

We defined surgery as any intervention that changes anatomy (including use of an implant), requires a skin or mucosal incision, or suturing to achieve the therapeutic effect. Surgical placebo was defined as an imitation procedure that cannot be differentiated by the patient but lacks the key therapeutic element.

We identified eligible placebo surgical studies from a published systematic review^2^ and extended the search to November 21, 2018. The reference lists of included studies were also checked for eligible studies. To identify relevant effectiveness trials, we first identified relevant Cochrane reviews assessing the index surgical procedure and extended their literature searches to March 13 to March 15, 2019. When no relevant Cochrane review was identified, we devised a search algorithm and searched from the inception of databases until the date of search (eAppendix 1 in the Supplement).

Searches were performed in the Cochrane Central Register of Controlled Trials (CENTRAL), MEDLINE and Embase databases. Pairs of authors (T.K., J.H., S.A., L.H., J.N., A.L., and J.J.) independently conducted the title and abstract screening, followed by full-text review to determine eligibility.

### Data Extraction

Pairs of authors (T.K., J.H., S.A., L.H., J.N., A.L., and J.J.) independently extracted the data. We extracted the following characteristics from each study: (1) study population; (2) sample size; (3) condition for which surgery was performed; (4) type of surgery (open or minimally invasive); (5) primary outcome; and (6) whether success of blinding was considered. Pairs of authors assessed the risk of bias for each study using the Cochrane Risk of Bias Tool v. 1.0.^14^ All discrepancies were solved by discussion.

### Choosing and Extracting Outcome Estimates

Depending on the study design, we extracted 2 or 3 within-group changes from each trial: (1) the observed change in the active surgery group (total effect of surgery); (2) observed change in the placebo surgery control group (total non-specific effects); and (3) observed change in the nonoperative control group (Figure 1 and Table 1).

We used the same type of outcome from corresponding studies (ie, same condition and intervention) whenever possible, prioritizing primary outcomes and time point from placebo-controlled studies. Because the primary outcome sometimes varied in otherwise similar placebo-controlled studies, we created a preferential hierarchy a priori: (1) a measure of pain; (2) function; (3) disease-specific quality of life; or (4) generic quality of life. To mitigate possible bias related to our choice of outcome, we performed sensitivity analyses with the prespecified primary outcomes in the included trials. When the time point was not defined or was irregular across studies, we selected a time point that likely reflected the timing of the maximum benefit of the surgical intervention being assessed.

### Data Handling and Statistical Analyses

We extracted the mean (SD) change within each group. If change scores were not reported, we calculated change using baseline and follow-up means and SDs.^15^ We used the median (IQR) as an approximation of mean when it was singularly reported. When unavailable, we estimated SD using 95% CIs, reported *P* values, (IQR) divided by 1.35 or range (range divided by 4).^16^ If we could not identify any data to estimate SD, we imputed SD from corresponding studies using the same outcome.

For binary outcomes, we extracted data on the number of participants classified as improved by the study authors (ie, a good outcome). Odds ratios (ORs) for change in binary outcomes were calculated as a ratio of the number of patients with a good outcome at follow up, to the number of patients with a good outcome at baseline.^17^ To enable the calculation of an OR in the presence of a cell with 0 counts (patients with a good outcome at baseline), 0.5 was added to each cell. ORs were log-transformed and converted into effect sizes.^18^

We harmonized the direction of effect so that positive within-group change indicated improvement and positive between-group effect size indicated a better outcome for the active surgery group. The treatment effects (between-group differences) were based on the difference in the amount of change from baseline and expressed as Hedges *g*. Treatment effects were calculated for active vs placebo surgery; active surgery vs nonoperative; placebo vs nonoperative.

To estimate the proportion of non-specific effects of the total change after surgery (including the placebo effect), we calculated the proportion for each placebo-controlled study as within-group change in the placebo surgical group divided by within-group change in the active surgical group. In pooling, we used the log of the proportion.^19^ We also pooled the within-group improvements in placebo groups (estimate for the magnitude of total nonspecific effects) in a separate meta-analysis. To estimate the magnitude of the surgical placebo effect, we pooled the differences between placebo and nonoperative control groups in studies that included both arms.

To estimate the association between type of the control group (placebo vs nonoperative) and the treatment effect, we performed metaregression using type of control as an effect modifier. Only those condition and interventions that had both placebo and nonoperative controlled studies were included. To see how other study characteristics impacted our analysis, we performed an additional analysis adjusting for possible differences in study characteristics, including sample size, subjective vs objective outcomes, and risk of bias (ie, high or unclear vs low for allocation concealment, attrition, and selective reporting).

Data were pooled using DerSimonian and Laird random effects meta-analyses. When 1 group contributed twice to the same meta-analysis, we halved the sample size for the active group.^16^

We assessed statistical heterogeneity using visual inspection and *I^2^* statistics. To assess heterogeneity of the metaregression analysis, we calculated the subgroup (placebo vs nonoperative control) differences in each condition and pooled these in a random effect meta-analysis. Significant statistical heterogeneity was defined as *I^2^* greater than 60% and *P* < .05. For publication bias, we performed an Egger test and assessed funnel plots when 10 or more studies were available for a condition and intervention group. Tests were 2-tailed, and analyses were conducted using STATA statistical software version 12.1 (StataCorp).

## Results

### Characteristics of Included Studies

We included data from 100 randomized controlled trials: 62 (62%) with placebo controls and 38 (38%) with nonoperative controls assessing the same surgical intervention for the same conditions as the placebo-controlled trials. Three trials included both placebo and nonoperative controls (eFigure 1 in the Supplement). Risk of bias was comparable across trials except for the risk of detection and performance bias which was high in the trials with nonoperative controls (Table 2; eTable 1 in the Supplement).

**Table 2.** Characteristics of Included Studies

Characteristics	Studies, No. (%)	
	Placebo-controlled	Nonoperative
Year of publication, median (IQR)	2011 (2005-2014)	2012 (2005-2014)
Studies^a^	62	41
Participants^a^	6122	4685
Participants/trial, median (IQR)	66 (43-130)	88 (53-139)
Proportion of females, %	68	60
Proportion of males, %	32	40
Self-reported outcome	47 (76)	35 (83)^b^
Proportion mini-invasive	55 (87)	37 (90)
Defined primary outcome	47 (76)	29 (71)
Low risk of allocation concealment	41 (65)	21 (51)
Low risk of attrition bias	52 (83)	28 (68)
Low risk of selective reporting	26 (41)	14 (34)
Measured success of blinding	13 (21)	NA

^a^ Studies that had both placebo and nonoperative controls are tallied in both columns. Studies that were excluded from the meta-analyses are not included in these numbers.

^b^ Alkatout et al (eAppendix 2 in the Supplement) were included in 2 condition-intervention pairs and measured both objective (ie, pregnancy) and subjective (ie, pain) outcomes.

The included trials assessed 32 different interventions or conditions in 10 699 randomized participants. For 15 intervention and condition pairs, we had both placebo-controlled trials (n = 31) and nonoperative controlled trials (n = 38) with a total of 8089 participants (eTable 2 in the Supplement). Details of trials that contributed to each analysis are included in eAppendix 2 in the Supplement.

Outcomes of included trials were pain (52 trials [52%]), function-related scores (32 [32%]); weight loss (6 [6%]); pregnancy (5 [5%]); and presence of stress urinary incontinence after pelvic prolapse surgery (4 [4%]) (eTable 2 in the Supplement). The median (IQR) time point of outcome data extraction was 12 (3-12) months. Log transformation of a binary outcome to calculate standardized mean difference (SMD) was performed in 27 (27%) trials. The remainder reported continuous outcomes.

### The Mean Proportion of Nonspecific Effects

**Figure 2.**
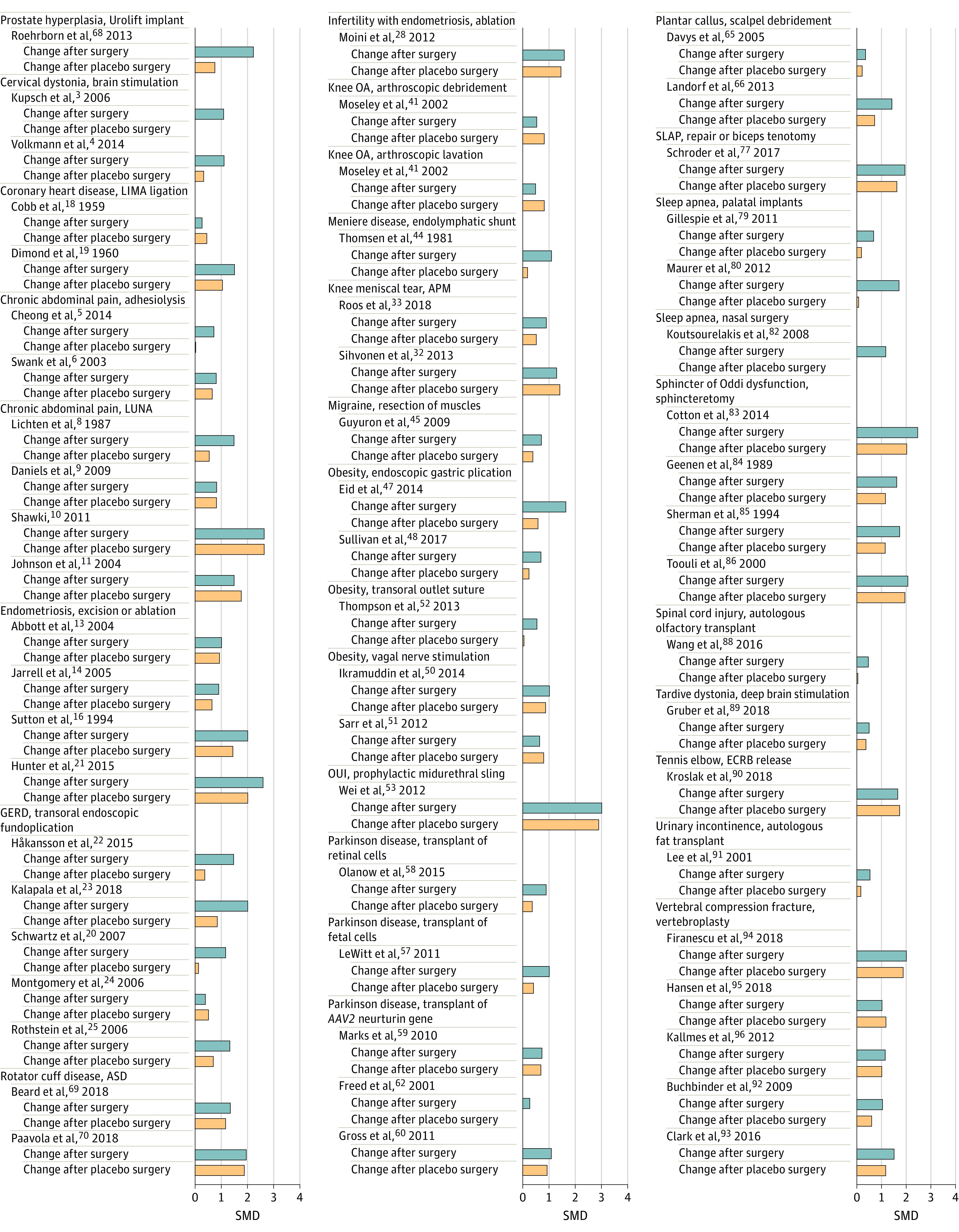
Observed Within Group Effect Sizes (Hedges *g*) in the Included Placebo-Controlled Trials in the Active Surgery and Placebo Surgery Groups APM indicates arthroscopic partial meniscectomy; ASD, arthroscopic subacromial decompression; ECRB, extensor carpi radialis brevis; GERD, gastroesophageal reflux disease; LIMA, left internal mammary artery; LUNA, laparoscopic uterosacral nerve ablation; OA, osteoarthritis; OUI, occult urinary incontinence; SLAP, superior labrum tear anterior to posterior. The reference numbers refer to eAppendix 2 in the Supplement full list of included studies.

The mean within group improvement in placebo arms (ie, total nonspecific effects) was SMD 0.74 (95% CI, 0.59 to 0.89; *I^2^* = 80%). As a proportion of the total effect of surgery, total nonspecific effects contributed between 0.01 and 1, and the mean pooled proportion was 0.67 (95% CI, 0.61 to 0.74; median [IQR], 0.71 [0.35-0.94]) (Figure 2; eTable 3 in the Supplement). The sensitivity analysis showed a mean pooled proportion of 0.73 (95% CI, 0.67 to 0.80; median [IQR], 0.75 [0.41-0.95]) using the primary outcomes defined by the trialists. Heterogeneity varied greatly between various interventions (*I^2^* = 0% to 100%) (eTable 3 in the Supplement).

### Magnitude of the Surgical Placebo Effect

In 3 trials with 556 participants that had both a placebo and nonoperative control,^20,21,22^ the pooled difference between these groups was SMD 0.13 (95% CI, −0.26 to 0.51; *I^2^* = 63%). In the sensitivity analysis, the pooled difference was similar (SMD, 0.10; 95% CI, −0.26 to 0.46).

### Difference in the Effect of Surgery Between Placebo- and Nonoperative-Controlled Trials

**Figure 3.**
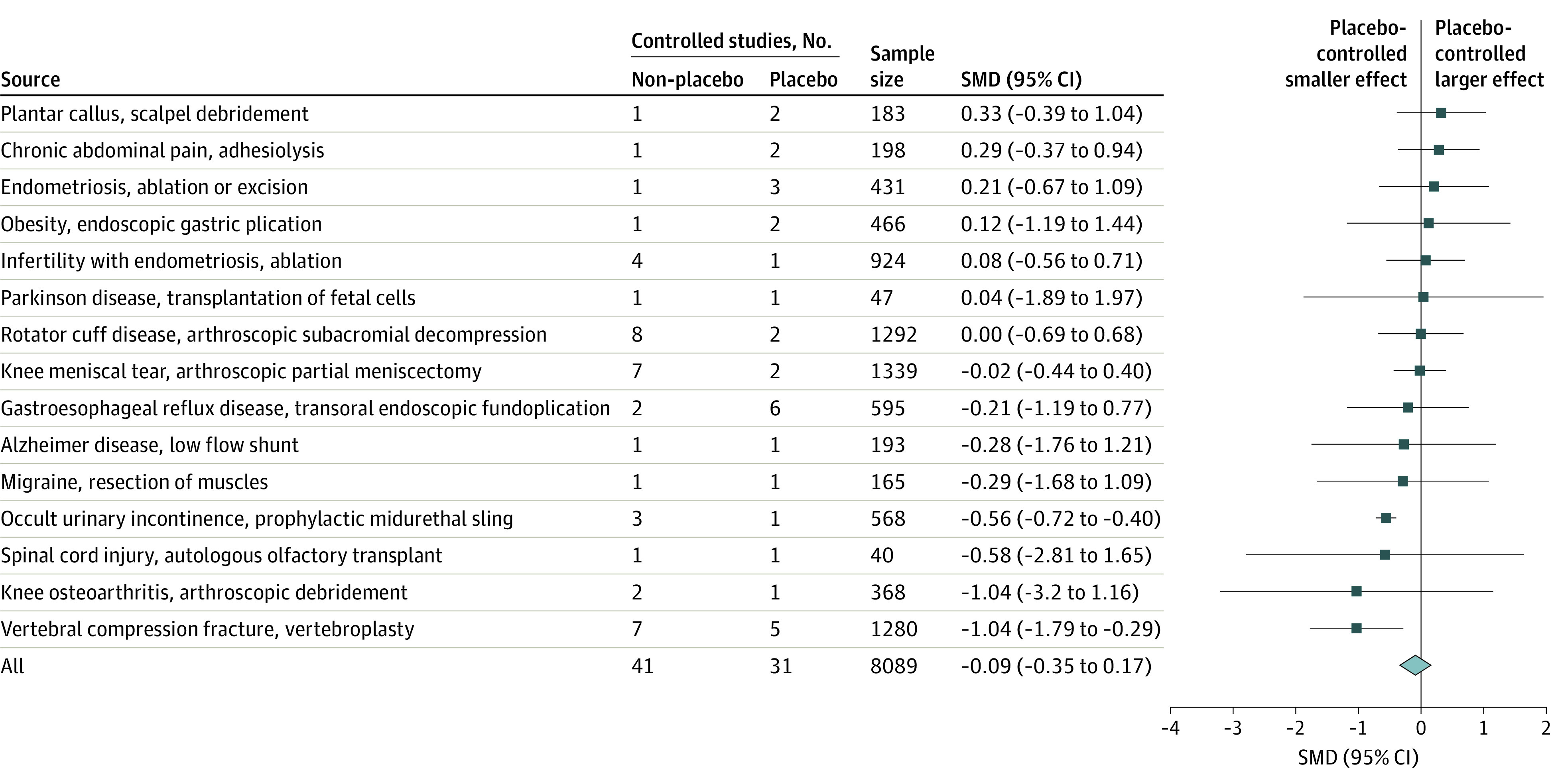
Subgroup Differences Between Placebo-Controlled and Non–Placebo Controlled Trials The effect of surgery was significantly smaller in placebo-controlled trials of vertebroplasty and prophylactic sling for urinary incontinence.

Metaregression included 73 between-group (active vs control) effects from 31 placebo-controlled trials and 38 trials with nonoperative controls. Type of control was not associated with the treatment effect. The mean difference (MD) between treatment effect in trials with a placebo control compared with those with nonoperative controls was −0.09 (95% CI, −0.35 to 0.17). The negative value indicates that placebo-controlled studies reported smaller effects than trials with nonoperative controls (Figure 3; eTable 3 in the Supplement). In the sensitivity analysis, the MD was −0.14 (95% CI, −0.41 to 0.14), the adjusted metaregression showed an SMD of −0.11 (95% CI, −0.37 to 0.15), and the sensitivity data set SMD of −0.18 (95% CI, −0.47 to 0.12).

Substantial heterogeneity was observed for the effect of surgery (*I^2^* = 89%), but the type of control group did not explain this heterogeneity. It was most likely explained by the different surgical interventions among the included studies. Meta-analysis of subgroup differences showed no statistical heterogeneity (*I^2^* = 0%) (Figure 3).

For 2 surgical interventions (vertebroplasty [12 trials] and prophylactic urethral sling [4 trials]), trials with nonoperative controls found larger benefits compared with placebo-controlled trials. Two conditions had the prerequisite number of studies (10 or more) for a separate metaregression. The SMD in the between-group effect size between placebo-controlled and nonoperative-controlled trials of subacromial decompression was 0 (95% CI, −0.8 to 0.8; 10 studies), and in vertebroplasty, it was 1.0 (95% CI, 0.18 to 1.9; 12 studies). The regression test for funnel plot asymmetry (performed in conditions with at least 10 trials) suggested no publication bias for studies within vertebroplasty (*P* = .9) or subacromial decompression trials (*P* = .65).

## Discussion

### Summary of Principal Findings

In this review, we did not find evidence supporting the hypothesis that surgery is associated with a large placebo effect. Trials with nonoperative controls were not associated with systematically larger effects of surgery compared with those using placebo controls. In studies that include both types of control, placebo groups did not improve more when compared with nonoperative controls.

Two-thirds of the total effects of surgery within the included trials comprised nonspecific effects. The estimated magnitude of the surgical placebo effect was only one-seventh of the total nonspecific effects; the metaregression (an indirect estimate of surgical placebo effect) suggested a similarly small placebo effect. This indicates that, in surgical placebo-controlled trials conducted to date, the large changes seen after placebo surgery^23^ occur mainly because of other nonspecific effects (ie, regression to the mean and natural course of disease).

It is difficult to define or measure the placebo effect precisely,^24^ but the important question is whether trialists should choose nonoperative or placebo controls for trials assessing the effect of surgery. Trials with nonoperative controls did not systematically overestimate effects of surgery (ie, the surgical placebo effect is not large enough to substantially bias the results). We found significant discrepancies in results between placebo and nonoperative controlled trials for only 2 interventions: vertebroplasty for osteoporotic vertebral fractures, where the primary outcomes were measured relatively early following the procedure (1 month), and urethral sling for occult urinary incontinence.

According to studies examining psychological mechanisms of the placebo effect, the placebo effect is based on expectations created by conditioning, verbal, or social cues.^25,26,27^ Conditioning plausibly does not occur in a single shot therapy, such as surgery, but regardless, conditioning may exist based on earlier experiences of receiving care. However, the theoretical model of conditioning predicts that the response will disappear if it is not maintained by new stimuli from receiving care.^28,29^ Pavlov’s dogs stopped salivating without new stimuli, and we hypothesize that people do not keep feeling better because they received a single conditioned signal (eg, surgery) months or years ago. A washout of the placebo effect is also supported by empirical findings. In drug experiments, the placebo effect disappears just like the effect of a drug within hours after administration,^30^ and in acupuncture studies, the placebo effect peaked at 12 weeks and disappeared by 52 weeks.^31^

### Findings in Respect to Existing Evidence

Like previous studies, we found large improvements after placebo surgery.^7,8,12,23^ However, this observed improvement should not be considered the placebo effect because it includes all nonspecific effects. Our analyses suggest that these improvements are largely related to factors other than the placebo effect.

Previous evidence on participant blinding through placebo procedure is conflicting. A systematic review^32^ assessing the effect of participant blinding found a mean difference of 0.37 SMD for subjective outcomes but with 95% CIs consistent with a null effect (−0.77 to 0.04). A recent meta-epidemiological study^33^ pooling data from 18 Cochrane reviews (138 trials) found no evidence that blinding affects the estimates (ratio of odd ratios [ROR], 0.98). Our study looking specifically at placebo controls in surgery supports these findings.

Contrary evidence includes the BRANDO study, which pooled data from 51 meta-analyses (530 trials), where the authors found a ROR of 0.78 (95% credible intervals, 0.65-0.92) for subjective outcomes suggesting that blinding attenuates the effect size.^34^ The Risk of Bias in Evidence Synthesis study pooled data from 81 meta-analyses (1017 trials) and found a similar ROR of 0.83 (95% credible intervals, 0.73-0.93).^35^ These studies suggest that bias may occur, but they did not assess surgery. Regarding our findings of nonspecific effects, our results are in line with a previous study assessing the nonspecific effects of 12 different mini-invasive procedures.^12^

### Limitations

This study had limitations. The relatively small number of published placebo-controlled surgical trials and heterogeneity in the magnitude of nonspecific effects across the included trials limit the certainty of our estimates. Based on the results, we cannot reject the existence of clinically important placebo effects for all types of surgery. The observed large proportion of nonspecific benefits is likely related to both the conditions and the interventions being assessed using a placebo-controlled trial design. It is likely that surgical treatments of doubtful efficacy, coupled with conditions that have a self-limiting natural course, are the ones most likely to be evaluated in placebo-controlled trials and thus meet our inclusion criteria. For example, we did not find placebo-controlled trials investigating surgical excision of malignant skin tumors which would otherwise continue to grow.

Various nonoperative cointerventions in the control arms in nonoperative controlled studies may confound our analyses but do not alter our interpretation of the findings that nonoperative controlled studies should not be routinely considered biased because of the surgical placebo effect. Although nonoperative comparators often contained active cointerventions (eg, physical therapy, injections, etc), surgery is seldom used alone, and postoperative protocols included similar components to control arms. We specifically excluded studies in which the nonoperative interventions were not plausibly available for the surgical group to minimize bias arising from nonoperative interventions.

The small number of studies including both placebo and nonoperative controls limits the certainty of the estimate of magnitude of surgical placebo effect. In theory, this comparison may overestimate the placebo effect. Participants not receiving placebo surgery are aware of the allocation and may not similarly adhere to postoperative rehabilitation and may report worse outcomes because of having lower expectations.^24^

By using the comprehensive searches of corresponding Cochrane reviews, we likely identified all relevant studies, but it is still possible we missed eligible trials. Our search was conducted in March 2019 but given our research question was related to the use of surgical placebo (rather than a clinical research question), it is unlikely additional recent trials will affect our conclusion.

## Conclusions

The findings of this systematic review suggest that although the difference in the effect size between placebo-controlled and nonoperative controlled trials was small, placebo-controlled trials yield the least biased results. However, a difference of SMD equal to 0.09 translates to only 0.2 points in pain measured on a 0 to 10 scale (using a typical SD of 2); and the upper boundary of the 95% CI (0.35 corresponds with 0.7 of 10 points) is well below what is considered clinically meaningful. When surgical trials with nonoperative controls have demonstrated consistently that a particular surgery does not provide clinically meaningful benefits, placebo-controlled trials are redundant. It is implausible that absence of a placebo control would hide a clinically important effect. However, as shown in the vertebroplasty and prophylactic sling for urinary incontinence trials, placebo-controlled trials are important when nonblinded trials show inconsistent results or the evidence suggests that the benefits may be small. Finally, the lack of a large placebo effect in surgery means that the use of ineffective surgical interventions for a placebo effect cannot be justified.

## Notes

Supplement. **eFigure.** PRISMA Flowchart **eTable 1.** Risk of Bias Judgements **eTable 2.** Conditions, Interventions, Number of Studies, the Extracted Outcomes, and Timepoints Where Outcomes Were Extracted **eTable 3.** Proportion of Nonspecific Effects for Each Study and Condition or Intervention Subgroup **eTable 4.** Effect Sizes in Studies Used in Meta-Regression Comparing Placebo and Nonplacebo Studies **eAppendix 1.** Search Strategies **eAppendix 2.** List of Included Trials
