# Supplementary material for: Use of Placebo and Nonoperative Control Groups in Surgical Trials: A Systematic Review and Meta-analysis
Source: JAMA Netw Open. 2022 Jul 27;5(7):e2223903. doi: 10.1001/jamanetworkopen.2022.23903 (PMC9331086; doi:10.1001/jamanetworkopen.2022.23903)
Supplement: Supplement. — eFigure. PRISMA Flowchart eTable 1. Risk of Bias Judgements eTable 2. Conditions, Interventions, Number of Studies, the Extracted Outcomes, and Timepoints Where Outcomes Were Extracted eTable 3. Proportion of Nonspecific Effects for Each Study and Condition or Intervention Subgroup eTable 4. Effect Sizes in Studies Used in Meta-Regression Comparing Placebo and Nonplacebo Studies eAppendix 1. Search Strategies eAppendix 2. List of Included Trials [file jamanetwopen-e2223903-s001.pdf]

Supplemental Online Content

Karjalainen T, Heikkinen J, Busija L, et al. Use of placebo and nonoperative control groups in surgical trials: a systematic review and meta-analysis. *JAMA Netw Open*. 2022;5(7):e2223903. doi:10.1001/jamanetworkopen.2022.23903

eFigure. PRISMA Flowchart
eTable 1. Risk of Bias Judgements
eTable 2. Conditions, Interventions, Number of Studies, the Extracted Outcomes, and Timepoints Where Outcomes Were Extracted
eTable 3. Proportion of Nonspecific Effects for Each Study and Condition or Intervention Subgroup
eTable 4. Effect Sizes in Studies Used in Meta-Regression Comparing Placebo and Nonplacebo Studies
eAppendix 1. Search Strategies
eAppendix 2. List of Included Trials

This supplemental material has been provided by the authors to give readers additional information about their work.

© 2022 Karjalainen T et al. *JAMA Network Open.*

eFigure. PRISMA Flowchart

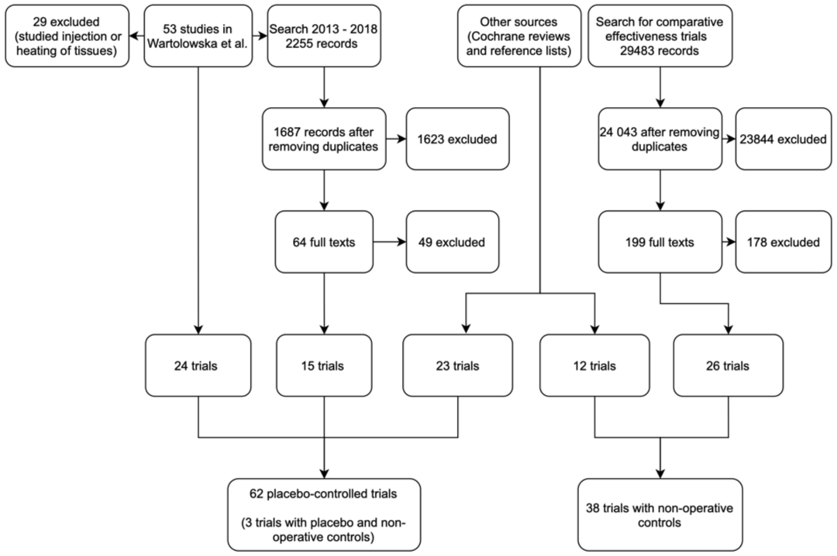

© 2022 Karjalainen T et al. *JAMA Network Open.*

eTable 1. Risk of Bias Judgements

Study	Year	Random sequence	Allocation concealment	Performance bias	Detection bias	Attrition bias	Selective reporting	Other
Abbott	2004	low	low	low	low	low	unclear	low
Alkatout a	2013	unclear	unclear	high	high	high	unclear	low
Beard non-placebo	2018	low	low	high	high	low	low	high
Beard Placebo	2018	low	low	low	low	low	low	low
Blasco	2012	low	unclear	high	high	unclear	unclear	low
Brox	1993	unclear	unclear	high	high	low	unclear	high
Buchbinder	2009	low	low	low	low	low	low	low
Chen a	2014	unclear	unclear	high	high	high	unclear	low
Chen b	2014	unclear	unclear	high	high	low	unclear	unclear
Cheong	2014	low	low	low	low	low	unclear	unclear
Clark	2016	low	low	unclear	low	unclear	high	unclear
Cobb	1959	unclear	low	unclear	low	low	unclear	low
Cotton	2014	unclear	unclear	low	low	low	low	low
Daniels	2009	low	low	unclear	low	high	unclear	low
Davys	2005	unclear	low	unclear	low	low	unclear	low
Dimond	1960	unclear	unclear	low	low	low	unclear	low
Eid	2014	low	low	unclear	low	high	low	high
Farfaras	2016	unclear	unclear	high	high	high	unclear	high
Farrokhi	2011	low	low	high	high	low	low	low
Firanescu	2018	low	low	low	low	low	high	low
Freed	2001	unclear	unclear	low	low	low	low	low
Friedman	2008	unclear	low	low	low	low	unclear	unclear
Fuentes	2011	unclear	low	high	high	low	high	low
Gad	2012	unclear	unclear	high	high	low	unclear	low
Gauffin	2014	low	low	high	high	low	low	low
Geenen	1989	unclear	low	low	low	low	high	low
Gillespie	2011	unclear	unclear	low	low	low	high	high
Gross	2011	unclear	low	low	low	low	low	low
Gruber	2018	unclear	unclear	unclear	low	low	low	unclear
Guyuron	2005	unclear	unclear	high	high	low	unclear	low

© 2022 Karjalainen T et al. *JAMA Network Open.*

Study	Year	Random sequence	Allocation concealment	Performance bias	Detection bias	Attrition bias	Selective reporting	Other
Guyuron	2009	low	low	low	low	low	high	unclear
Haahr	2005	low	low	high	high	low	high	low
Hansen	2016	low	low	low	low	low	unclear	unclear
Herrlin	2007	low	low	high	high	low	high	low
Hunter	2015	low	low	low	low	low	low	high
Håkansson	2015	low	low	unclear	low	low	low	low
Ikramuddin	2014	unclear	unclear	low	low	low	low	low
Jarrell	2005	low	low	low	low	high	unclear	low
Johnson	2004	low	low	low	low	low	unclear	unclear
Kalapala	2018	low	unclear	low	low	unclear	unclear	low
Kallmes	2009	low	low	low	low	low	low	low
Katz	2013	low	low	high	high	low	high	unclear
Ketola	2009	low	low	high	high	high	unclear	unclear
Kirkley	2008	low	low	high	high	high	low	low
Klazen	2010	low	low	high	high	unclear	low	unclear
Koutsourelarakis	2008	low	unclear	low	low	low	unclear	low
Kroslak	2018	low	low	low	low	low	unclear	unclear
Kupsch	2006	unclear	unclear	unclear	low	low	low	low
Landorf	2013	unclear	low	low	low	low	low	low
Lee	2001	low	low	low	low	unclear	unclear	unclear
LeWitt	2011	low	low	low	low	high	unclear	low
Lichten	1987	high	high	unclear	low	low	unclear	low
Marcoux	1997	low	low	high	high	low	unclear	low
Marks	2010	low	unclear	low	low	low	high	unclear
Maurer	2012	unclear	unclear	low	low	low	low	low
Merchan	1993	unclear	unclear	high	high	unclear	unclear	low
Miller	2017	unclear	unclear	high	high	low	unclear	low
Moini	2012	unclear	low	high	low	high	unclear	low
Montgomery	2006	unclear	low	low	low	low	unclear	low
Moseley (debridement)	2002	unclear	low	low	low	low	low	low
Moseley (lavation)	2002	unclear	low	low	low	low	low	low
Olanow	2015	low	unclear	low	low	low	low	low
Olanow	2003	low	unclear	low	low	low	high	low
Osterås	2012	unclear	unclear	high	high	low	unclear	unclear

© 2022 Karjalainen T et al. *JAMA Network Open.*

Study	Year	Random sequence	Allocation concealment	Performance bias	Detection bias	Attrition bias	Selective reporting	Other
Paavola non-placebo	2018	low	low	high	high	low	low	low
Paavola placebo	2018	low	low	low	low	low	low	low
Parrazini	1999	low	low	high	high	low	unclear	low
Peters	1992	unclear	unclear	high	high	low	high	low
Peters	1997	unclear	unclear	high	high	unclear	unclear	low
Rahme	1998	unclear	unclear	high	high	unclear	high	high
Roehrborn	2013	low	low	low	low	low	low	low
Roos	2018	low	low	low	low	low	low	high
Rothstein	2006	low	low	unclear	low	low	low	unclear
Rousing	2010	unclear	unclear	high	high	high	unclear	unclear
Sarr	2012	unclear	unclear	low	low	low	unclear	low
Schierlitz	2014	low	unclear	high	high	low	low	low
Schroder	2017	unclear	low	low	low	low	unclear	high
Schwartz	2007	unclear	low	low	low	low	low	low
Schwartz non-placebo	2007	unclear	low	high	high	low	low	low
Shawki	2011	low	low	unclear	low	low	unclear	low
Sherman	1994	unclear	unclear	unclear	unclear	unclear	unclear	unclear
Siddle	2013	low	low	high	high	low	high	unclear
Sihvonen	2013	low	low	low	low	low	unclear	low
Silverberg	2002	low	low	high	high	low	unclear	unclear
Silverberg	2008	low	low	low	low	high	high	low
Spencer	1992	unclear	unclear	high	high	low	unclear	unclear
Stensrud	2015	low	low	high	high	low	low	low
Steward	2008	low	low	low	low	low	high	unclear
Sullivan	2017	unclear	unclear	low	low	unclear	low	low
Sutton	1994	low	unclear	unclear	low	low	unclear	low
Swank	2003	low	low	unclear	low	low	unclear	low
Thompson	2013	low	low	low	low	low	low	high
Thomsen	1981	unclear	unclear	unclear	low	low	high	low
Toouli	2000	unclear	unclear	low	low	low	unclear	low
Trad	2015	low	low	high	high	low	low	unclear
van der Ploeg	2016	low	low	high	high	low	low	low
VandeGraaf	2018	low	low	high	high	low	low	low
Volkman	2014	unclear	low	high	low	low	low	low

© 2022 Karjalainen T et al. *JAMA Network Open.*

Study	Year	Random sequence	Allocation concealment	Performance bias	Detection bias	Attrition bias	Selective reporting	Other
Voormoolen	2006	unclear	unclear	high	high	unclear	unclear	low
Wang	2016	unclear	unclear	low	low	low	high	low
Wei	2012	low	unclear	low	low	low	low	low
Witteman	2015	low	low	high	high	low	low	low
Yang	2016	low	unclear	high	high	high	unclear	low
Yim	2013	unclear	low	high	high	low	high	unclear

© 2022 Karjalainen T et al. *JAMA Network Open.*

eTable 2. Conditions, Interventions, Number of Studies, the Extracted Outcomes, and Timepoints Where Outcomes Were Extracted

**Condition**	**Intervention**	**Interventions in non-blinded trials**	**Number of placebo studies**	**Number of non-blinded studies**	**Outcome 1)In the primary analysis 2)In the sensitivity analysis**	**Timepoints where effects were extracted**
Alzheimer's disease	Low-flow-ventriculo-peritoneal shunt	Shunt versus no treatment	1	1	1)Mattis Dementia Rating Scale<br>2)Same as primary	9 months<br>12 months
Cervical dystonia	Deep brain stimulation	N/A	2	0	1) Pain, TWSTRS score[a]<br>2) BFM score[b], TWSTRS score[a]	3 months
Chronic abdominal pain	Adhesiolysis	Adhesiolysis versus usual care	2	1	1) Pain<br>2) Same as primary	11-12 months (2 studies)<br>6 months (1 study)
Chronic abdominal or pelvic pain	Laparoscopic uterosacral nerve ablation	N/A	4	0	1) Pain (2 continuous, 2 binary)<br>2) Same as primary	3 months (1 study)<br>6 months (4 studies)
Chronic abdominal or pelvic pain with endometriosis	Excision/ablation	Laparoscopic excision and leuprorelin versus leuprorelin alone	4	1	1) Pain<br>2) Same as primary	6 months (4 studies)<br>3 months (1 study)
Coronary heart disease	Left internal mammary artery ligation	N/A	2	0	1) Subjective improvement, exercise tolerance<br>2) Same as primary	3-15 months
Gastroesophageal reflux disease	Transoral endoscopic fundoplication	Transoral endoscopic fundoplication with proton pump inhibitor medication versus medication only	6	3	1) Success (3 studies),GERD HRQL[c] (4 studies), GSRS score[d] (1 study), Symptom score (1 study)<br>2) Same as primary	3 months (2 studies)<br>6 months (4 studies)<br>12 months (1 study)
Infertility with endometriosis	Ablation	Excision/ablation vs usual care	1	4	Pregnancy rate	8-18 months
Knee meniscal tear	Arthroscopic partial meniscectomy	Arthroscopic meniscectomy versus exercise therapy	2	7	KOOS[e] pain subscore (5 studies), VAS[f] pain (3 studies), VAS after exercise (1 study)	12 months (7 studies)<br>24 months (1 study)
Knee osteoarthritis	Arthroscopic debridement	Arthroscopic debridement versus exercise and medical therapy	1	2	KOOS pain, WOMAC[g] pain subscore	12 months
Knee osteoarthritis	Arthroscopic lavation	N/A	1	0	Womac[g] pain subscore	12 months
Meniere	Endolymphatic shunt		1	0		
Migraine	Resection of muscles[h]	Removal of muscles versus usual care	1	1	1) Migraine headache index<br>2) Migraine headache index	12 months
Obesity	Transoral gastric fundoplication [g]	Endoscopic gastric plication versus usual care	2	1	1) Percent weight loss (2 studies), Number achieving 15% excess weight loss (1 study)<br>2) Same as primary	6 months (1 study)<br>12 months (2 studies)

© 2022 Karjalainen T et al. *JAMA Network Open.*

Condition	Intervention	Interventions in non-blinded trials	Number of placebo studies	Number of non-blinded studies	Outcome<br>1)In the primary analysis<br>2)In the sensitivity analysis	Timepoints where effects were extracted
Obesity	Vagal nerve stimulation	Vagal nerve stimulation versus usual care	2	0	1) Percent weight loss<br>2) Same as primary	12 months
Obesity	Transoral outlet suture	N/A	1	0	Percent weight loss	
Occult urinary incontinence	Prophylactic midurethral sling	Prophylactic midurethral sling versus usual care	1	3	1) Number with no SUI[i] (2 studies), Secondary surgery for SUI[j] (2 studies)<br>2) Same as primary	6 months (1 study)<br>12 months (2 studies)<br>20 months (1 study)
Parkinson's disease	Transplantation of AAV2 neurturin gene	N/A	4	0	1) Off-medication UPDRS[k] score (4 studies)<br>2) Same as primary	6 months (1 study)<br>12 months (1 study)<br>18 months (1 study)
Parkinson's disease	Transplantation of human retinal pigment epithelial cells	N/A	1	0	1) Off-mediation UPDRS[k] score<br>2) Same as primary	12 months
Parkinson's disease	Transplantation of fetal cells	Transplant versus usual care	2	1	1) Off-medication UPDRS[k] score<br>2) Same as primary	12 months (1 study)<br>18 months (1 study)
Plantar callus	Scalpel debridement	Scalpel + 'therapeutic approach' versus 'therapeutic approach' alone	2	1	1) Pain<br>2) Same as primary	4 -6 weeks (2 studies)<br>18 months (1 study)
Prostate hyperplasia	UroLift implant®	N/A	1	0	1) American Urological Association Symptom Index<br>2) Same as primary	3 months
Rotator cuff disease	Arthroscopic subacromial decompression	Arthroscopic subacromial decompression versus exercise therapy	2	8	1) Pain VAS/NRS (7 studies), Subjective shoulder Score (1 study), Constant Score (1 study)<br>2) Pain. OSS[l], Subjective shoulder score, Constant-Murley score	12 months (9 studies)<br>3 years (1 study)
SLAP[m] lesion	Repair or biceps tenotomy	N/A	1	0	1) Pain<br>2) Rowe score	24 months
Sleep apnea	Palatal implants	N/A	4	0	1) AHI index[n] (3studies), Sleep related Quality of life score (1 study)<br>2) Same as primary	3 months
Sleep apnea	Nasal surgery	N/A	1	0	1) AHI index[n]<br>2) Same as primary	3-4 months
Sphincter of oddi dysfunction	Sphincterotomy	N/A	4	0	1) Success[o] (1 study), Symptom score[p] (1 study), Improvement (2 studies)<br>2) Same as primary	9 to 12 months (2 studies)<br>2 years (1 study)<br>3 years (1 study)
Spinal cord injury	Autologous olfactory transplant	Autologous olfactory transplant versus rehabilitation/usual care	1	1	1) ASIA motor score[q], SCI-FRS score[r]<br>2) Same as primary	6 months (1 study)<br>36 months (1 study)

© 2022 Karjalainen T et al. *JAMA Network Open.*

Condition	Intervention	Interventions in non-blinded trials	Number of placebo studies	Number of non-blinded studies	Outcome<br>1)In the primary analysis<br>2)In the sensitivity analysis	Timepoints where effects were extracted
Tardive dystonia	Deep brain stimulation	N/A	1	0	BFM score[b]	3 months
Tennis elbow	ECRB[s] release	N/A	1	0	1) Pain improvement<br>2) Pain frequency	6 months
Vertebral compression fracture	Vertebroplasty	Vertebroplasty versus usual care	5	7	1) Pain (12 studies)<br>2) Same as primary	2 weeks (1 study)<br>1 month (8 studies)<br>3 months (3 studies)
		**TOTAL number[t]**	**65**	**41**		

[a] Toronto Western Spasmodic Torticollis Rating Scale
[b] Movement subscore on the Burke–Fahn–Marsden Dystonia Rating Scale
[c] Gastroesophageal reflux disease specific health-related quality of life
[d] Gastrointestinal Symptom Rating Scale
[e] Knee injury and Osteoarthritis Outcome Score
[f] Visual Analog Scale
[g] The Western Ontario and McMaster Universities Osteoarthritis Index
[h] Depending on the location of the headache removal of the glabellar muscles (corrugator supercilii, depressor supercilii, and procerus), or removal of a segment of the zygomaticotemporal branch of the trigeminal nerve or A segment of the semispinalis capitis muscle medial to the greater occipital nerve approximately 1 cm wide and 2.5 cm long.
[i] StomaphyX; or POSE-procedure (g-Cath EZ Delivery Catheter with Snowshoe Suture Anchors and its accessories); or transoral outlet reduction
[j] Urinary Stress Incontinence
[k] Unified Parkinson’s Disease Rating Scale (motor or total score)
[l] Oxford Shoulder Score
[m] Superior Labrum tear Anterior to Posterior (SLAP)
[n] Apnea Hypoxia Index= Number of apneas per hour of sleep
[o] Success = less than 6 days of disability due to pain in the prior 90 days both at months 9 and 12 after randomization, with no narcotic use and no further sphincter intervention
[p] Proportion of participants with symptom score to 0 to 2 at follow-up
[q] American Spinal Injury Association score
[r] Spinal Cord Injury Functional Rating Scale
[s] Extensor carpi radialis brevis
[t] Studies with both placebo and non-placebo controls are tallied in both columns

© 2022 Karjalainen T et al. *JAMA Network Open.*

eTable 3. Proportion of Nonspecific Effects for Each Study and Condition/Intervention Subgroup

**Intervention/Condition** Study id	**N Studies**	**N[a]**	**Total effect of surgery (SMD)**	**95% CI**		**I² for effect**	**Proportion of non-specific effect[b]**	**95% CI**		**I² for proportion**
**Brain stimulation/ Cervical dystonia**	2	102				0 %				98 %
Kupsch 2006			1.10	0.56	1.65		0.01	0.00	0.02	
Volkman 2014			1.12	0.68	1.55		0.31	0.25	0.37	
D+L pooled ES			*1.11*	*0.77*	*1.45*		*0.05*	*0.00*	*1.71*	
**Adhesiolysis/ Chronic abdominal pain**	2	150				0 %				72 %
Cheong 2014			0.73	0.28	1.18		0.04	0.00	0.89	
Swank 2003			0.82	0.51	1.13		0.82	0.75	0.89	
D+L pooled ES			*0.79*	*0.54*	*1.05*		*0.28*	*0.02*	*4.49*	
**LUNA[c]/ Chronic abdominal or pelvic pain**	4	710				56 %				47 %
Lichten 1987			1.50	0.05	2.95		0.37	0.14	0.98	
Daniels 2009			0.84	0.67	1.00		0.99	0.97	1.01	
Shawki 2011			2.65	1.13	4.17		1.00	0.92	1.09	
Johnson 2004			1.50	-0.01	3.02		1.18	0.90	1.54	
D+L pooled ES			*1.40*	*0.59*	*2.22*		*1.00*	*0.93*	*1.08*	
**LIMA[d] ligation/ Coronary heart disease**	2	35				57 %				58 %
Cobb 1959			0.28	-0.35	0.91		1.64	0.66	4.08	
Dimond 1960			1.52	0.05	2.99		0.70	0.35	1.38	
D+L pooled ES			*0.72*	*-0.44*	*1.87*		*1.00*	*0.43*	*2.28*	
**Transoral endoscopic fundoplication/ GERD[e]**	6	452				74 %				96 %
Hunter 2015			2.60	1.08	4.12		0.76	0.67	0.87	
Håkansson 2015			1.48	0.89	2.08		0.26	0.20	0.33	
Kalapala 2018			1.98	0.48	3.48		0.43	0.29	0.65	
Schwartz 2007			1.18	0.62	1.73		0.12	0.06	0.24	

© 2022 Karjalainen T et al. *JAMA Network Open.*

**Intervention/Condition**<br>Study id	**N Studies**	**N[a]**	**Total effect of surgery (SMD)**	**95% CI**		**I² for effect**	**Proportion of non-specific effect[b]**	**95% CI**		**I² for proportion**
Montgomery 2006			0.41	-0.01	0.83		1.28	0.98	1.69	
Rothstein 2006			1.34	1.00	1.68		0.53	0.50	0.56	
D+L pooled ES			*1.27*	*0.78*	*1.76*		*0.47*	*0.33*	*0.69*	
**Ablation or excision/ Endometriosis**	3	131				25 %				29 %
Abbott 2004			1.01	0.49	1.54		0.94	0.79	1.11	
Jarrell 2005			0.92	0.11	1.72		0.72	0.45	1.14	
Sutton 1994			2.00	0.49	3.51		0.73	0.58	0.92	
D+L pooled ES			*1.23*	*0.70*	*1.75*		*0.81*	*0.70*	*0.92*	
**Ablation/ Infertility with endometriosis**	1	146								
Moini 2012			1.59	0.05	3.13		0.92	0.73	1.16	
D+L pooled ES			*1.59*	*0.05*	*3.13*		*0.92*	*0.73*	*1.16*	
**Arthroscopic partial meniscectomy/ Knee meniscal tear**	2	190				44 %				97 %
Roos 2018			0.91	0.42	1.39		0.58	0.46	0.72	
Sihvonen 2013			1.30	0.98	1.62		1.09	1.05	1.14	
D+L pooled ES			*1.15*	*0.77*	*1.52*		*0.80*	*0.43*	*1.50*	
**Arthroscopic debridement/ Knee osteoarthritis**	1	117								
Moseley 2002			0.54	0.25	0.83		1.53	1.40	1.68	
D+L pooled ES			*0.54*	*0.25*	*0.83*		*1.53*	*1.40*	*1.68*	
**Arthroscopic lavation/ Knee osteoarthritis**	1	120								
Moseley 2002			0.50	0.22	0.77		1.67	1.53	1.82	
D+L pooled ES			*0.50*	*0.22*	*0.77*		*1.67*	*1.53*	*1.82*	
**Endolymphatic shunt/ Meniere**	1	30								
Thomsen 1981			1.10	0.48	1.72		0.17	0.09	0.34	
D+L pooled ES			*1.10*	*0.48*	*1.72*		*0.17*	*0.09*	*0.34*	

© 2022 Karjalainen T et al. *JAMA Network Open.*

**Intervention/Condition** Study id	**N Studies**	**N[a]**	**Total effect of surgery (SMD)**	**95% CI**		**I² for effect**	**Proportion of non-specific effect[b]**	**95% CI**		**I² for proportion**
**Resection of muscles/ Migraine**	1	75								
Guyuron 2009			0.72	0.41	1.03		0.55	0.45	0.67	
D+L pooled ES			*0.72*	*0.41*	*1.03*		*0.55*	*0.45*	*0.67*	
**Endoscopic gastric plication/Obesity**	2	422				31 %				0 %
Eid 2014			1.65	0.11	3.19		0.36	0.20	0.63	
Sullivan 2017			0.70	0.55	0.85		0.35	0.32	0.38	
D+L pooled ES			*0.85*	*0.17*	*1.54*		*0.35*	*0.32*	*0.38*	
**Vagal nerve stimulation /Obesity**	2	533				89 %				100 %
Ikramuddin 2014			1.03	0.84	1.21		0.86	0.83	0.89	
Sarr 2012			0.65	0.49	0.81		1.24	1.20	1.28	
D+L pooled ES			*0.83*	*0.47*	*1.20*		*1.03*	*0.72*	*1.48*	
**Transoral outlet suture/Obesity**	1	77								
Thompson 2013			0.55	0.25	0.84		0.10	0.03	0.37	
D+L pooled ES			*0.55*	*0.25*	*0.84*		*0.10*	*0.03*	*0.37*	
**Prophylactic midurethral sling/ Occult urinary incontinence**	1	337								
Wei 2012			3.01	1.49	4.53		0.96	0.91	1.02	
D+L pooled ES			*3.01*	*1.49*	*4.53*		*0.96*	*0.91*	*1.02*	
**Transplantation of AAV2 neurturin gene/Parkinson's disease**	3	154				0 %				95 %
Olanow 2015			0.90	0.43	1.37		0.41	0.32	0.52	
LeWitt 2011			0.99	0.41	1.57		0.42	0.32	0.55	
Marks 2010			0.74	0.39	1.10		0.94	0.79	1.12	
D+L pooled ES			*0.84*	*0.58*	*1.09*		*0.55*	*0.30*	*0.99*	

© 2022 Karjalainen T et al. *JAMA Network Open.*

Intervention/Condition<br>Study id	N Studies	N[a]	Total effect of surgery (SMD)	95% CI		I² for effect	Proportion of non-specific effect[b]	95% CI		I² for proportion
**Transplantation of human retinal pigment epithelial cells/Parkinson's disease**	1	71								
Gross 2011			1.10	0.68	1.51		0.85	0.78	0.94	
D+L pooled ES			*1.10*	*0.68*	*1.51*		*0.85*	*0.78*	*0.94*	
**Transplantation of fetal cells/Parkinson's disease**	1	40								
Freed 2001			0.28	-0.15	0.71		0.04	0.00	207	
D+L pooled ES			*0.28*	*-0.15*	*0.71*		*0.04*	*0.00*	*207*	
**Scalpel debridement/ Plantar callus**	2	118				91 %				0 %
Davys 2005			0.35	-0.10	0.81		0.61	0.35	1.07	
Landorf 2013			1.43	0.99	1.86		0.51	0.46	0.55	
D+L pooled ES			*0.89*	*-0.16*	*1.94*		*0.51*	*0.47*	*0.56*	
**Urolift implant/ Prostate hyperplasia**	1	206								
Roehrborn 2013			2.24	1.93	2.55		0.34	0.33	0.36	
**Arthroscopic subacromial decompression/ Rotator cuff disease**	2	331				80 %				93 %
Beard 2018			1.36	1.07	1.65		0.87	0.84	0.90	
Paavola 2018			1.97	1.52	2.42		0.96	0.92	1.00	
D+L pooled ES			*1.64*	*1.04*	*2.23*		*0.91*	*0.82*	*1.01*	
**Repair or biceps tenotomy/SLAP[f] lesion**	1	78								
Schroder 2017			1.95	1.42	2.48		0.83	0.78	0.89	
D+L pooled ES			*1.95*	*1.42*	*2.48*		*0.83*	*0.78*	*0.89*	
**Palatal implants/ Sleep apnea**	2	73				74 %				51 %

© 2022 Karjalainen T et al. *JAMA Network Open.*

**Intervention/Condition** Study id	**N Studies**	**N[a]**	**Total effect of surgery (SMD)**	**95% CI**		**I² for effect**	**Proportion of non-specific effect[b]**	**95% CI**		**I² for proportion**
Gillespie 2011			0.68	0.26	1.10		0.27	0.17	0.44	
Maurer 2012			1.72	0.78	2.66		0.04	0.00	0.54	
D+L pooled ES			*1.11*	*0.11*	*2.11*		*0.16*	*0.03*	*0.87*	
**Nasal surgery/Sleep apnea**	1	49								
Koutsourelarakis 2008			1.18	-0.39	2.74		0.01	0.00	0.74	
D+L pooled ES			*1.18*	*-0.39*	*2.74*		*0.01*	*0.00*	*0.74*	
**Sphincterotomy/ Sphincter of oddi dysfunction**	4	376				0 %				28 %
Cotton 2014			2.47	0.95	4.00		0.82	0.74	0.91	
Geenen 1989			1.62	0.11	3.13		0.72	0.52	1.01	
Sherman 1994			1.74	0.25	3.23		0.66	0.45	0.97	
Toouli 2000			2.08	0.57	3.59		0.94	0.79	1.11	
D+L pooled ES			*1.97*	*1.22*	*2.73*		*0.83*	*0.74*	*0.93*	
**Autologous olfactory transplant/Spinal cord injury**	1	12								
Wang 2016			0.47	-0.19	1.12		0.09	0.00	2919.01	
D+L pooled ES			*0.47*	*-0.19*	*1.12*		*0.09*	*0.00*	*2919.01*	
**Deep brain stimulation/Tardive dystonia**	1	25								
Gruber 2018			0.50	-0.07	1.06		0.74	0.44	1.23	
D+L pooled ES			*0.50*	*-0.07*	*1.06*		*0.74*	*0.44*	*1.23*	
**ECRB[g] release/Tennis elbow**	1	26								
Kroslak 2018			1.66	0.85	2.48		1.05	0.87	1.27	
D+L pooled ES			*1.66*	*0.85*	*2.48*		*1.05*	*0.87*	*1.27*	
**Autologous fat transplant/Urinary incontinence**	1	68								
Lee 2001			0.54	0.19	0.88		0.30	0.20	0.44	

© 2022 Karjalainen T et al. *JAMA Network Open.*

**Intervention/Condition** Study id	**N Studies**	**N[a]**	**Total effect of surgery (SMD)**	**95% CI**		**I² for effect**	**Proportion of non-specific effect[b]**	**95% CI**		**I² for proportion**
D+L pooled ES			*0.54*	*0.19*	*0.88*		*0.30*	*0.20*	*0.44*	
**Vertebroplasty/Vertebral compression fracture**	5	561				79 %				97 %
Firanescu 2018			2.00	1.64	2.36		0.94	0.92	0.97	
Hansen 2016			1.02	0.52	1.52		1.16	1.01	1.33	
Kallmes 2009			1.15	0.85	1.46		0.85	0.81	0.90	
Buchbinder 2009			1.05	0.64	1.45		0.58	0.52	0.65	
Clark 2016			1.51	1.13	1.90		0.77	0.73	0.81	
D+L pooled ES			*1.36*	*0.99*	*1.73*		*0.84*	*0.73*	*0.96*	
D+L pooled ES Overall			***1.10***	***0.96***	***1.23***		***0.67***	***0.61***	***0.74***	
Sensitivity dataset			***1.09***	***0.96***	***1.23***		***0.73***	***0.67***	***0.80***	

[a] Number randomised
[b] [Observed effect in the placebo surgery group] / [Observed effect in the active surgery group]
[c] Laparoscopic uterosacral nerve ablation
[d] Left Internal Mammary Artery
[e] GastroEsophageal Reflux Disease
[f] Superior Labrum tear Anterior to Posterior (SLAP)
[g] Extensor Carpi Radialis Brevis

© 2022 Karjalainen T et al. *JAMA Network Open.*

eTable 4. Effect Sizes in Studies Used in Meta-Regression Comparing Placebo and Nonplacebo Studies

		Non-placebo							Placebo				
**Intervention/ Condition**	**N Studies**	**N Observations**	**Effect size**	**95%CI**		**I²**	**Study**	**N Studies**	**N Observations**	**Effect size**	**95%CI**		**I²**
**Low flow shunt/ Alzheimer's disease**	1	29						1	164				
Silverberg 2002			0.47	-0.35	1.28		Silverberg 2008			0.19	-0.12	0.50	
Pooled ES			*0.47*	*-0.35*	*1.28*		*Pooled ES*			*0.19*	*-0.12*	*0.50*	
**Adhesiolysis/ Chronic abdominal pain**	1	48						2	150				5 %
Peters 1992			-0.03	-0.59	0.53		Cheong 2014			0.50	-0.10	1.09	
							Swank 2003			0.12	-0.27	0.51	
Pooled ES			*-0.03*	*-0.59*	*0.53*		*Pooled ES*			*0.24*	*-0.10*	*0.58*	
**Transoral endoscopic fundoplication /GERD[a]**	2	123				0 %		6	452				88 %
Trad 2015			0.95	0.40	1.50		Hunter 2015			0.48	0.25	0.70	
Witteman 2015			1.13	0.55	1.70		Håkansson 2015			1.09	0.47	1.71	
Schwartz 2007 non-placebo			0.86	0.08	1.64		Kalapala 2018			2.04	1.54	2.53	
							Schwartz 2007			1.07	0.42	1.72	
							Montgomery 2006			-0.14	-0.71	0.43	
							Rothstein 2006			0.76	0.41	1.11	
Pooled ES			*0.96*	*0.48*	*1.44*		*Pooled ES*			*0.87*	*0.35*	*1.39*	
**Ablation or excision/ Endometriosis**	1	300						3	131				62 %
Alkatout 2013			0.31	0.13	0.49		Abbott 2004			0.10	-0.52	0.71	
							Jarrell 2005			0.26	-0.68	1.20	
							Sutton 1994			0.91	0.59	1.24	
Pooled ES			*0.31*	*0.13*	*0.49*		*Pooled ES*			*0.52*	*-0.64*	*1.68*	
**Ablation/ Infertility with endometriosis**	4	778				88 %		1	146				
Alkatout 2013			-0.11	-0.26	0.03		Moini 2012			0.17	-0.16	0.49	
Parrazini 1999			-0.15	-0.42	0.12								

© 2022 Karjalainen T et al. *JAMA Network Open.*

		Non-placebo								Placebo				
**Intervention/ Condition**	**N Studies**	**N Observations**	**Effect size**	**95%CI**		**I²**	**Study**	**N Studies**	**N Observations**	**Effect size**	**95%CI**		**I²**
Gad 2012			0.28	-0.12	0.67								
Marcoux 1997			0.37	0.22	0.53								
Pooled ES			*0.09*	*-0.21*	*0.39*		*Pooled ES*			*0.17*	*-0.16*	*0.49*	
**Arthroscopic partial meniscectomy/ Knee meniscal tear**	7	1149				37 %		2	190				33 %
Stensrud 2015			0.14	-0.21	0.48		Roos 2018			0.38	-0.22	0.98	
Gauffin 2014			0.50	0.15	0.85		Sihvonen 2013			-0.04	-0.37	0.28	
Herrlin 2007			0.18	-0.23	0.59								
Katz 2013			0.02	-0.19	0.24								
Osterås 2012			-0.53	-1.45	0.39								
Yim 2013			-0.13	-0.52	0.25								
VandeGraaf 2018			0.21	-0.02	0.45								
Pooled ES			*0.13*	*-0.03*	*0.29*		*Pooled ES*			*0.09*	*-0.30*	*0.48*	
**Arthroscopic debridement/ Knee osteoarthritis**	2	251				97 %		1	117				
Kirkley 2008			0.14	-0.17	0.46		Moseley 2002			-0.25	-0.63	0.14	
Merchan 1993			1.44	1.10	1.78								
Pooled ES			*0.79*	*-0.48*	*2.06*		*Pooled ES*			*-0.25*	*-0.63*	*0.14*	
**Resection of muscles/Migraine**	1	108						1	75				
Guyuron 2005			0.72	0.22	1.22		Guyuron 2009			0.42	-0.05	0.90	
Pooled ES			*0.72*	*0.22*	*1.22*		*Pooled ES*			*0.42*	*-0.05*	*0.90*	
**Endoscopic gastric plication/ Obesity**	1	44						2	422				80 %
Miller 2017			0.43	-0.31	1.17		Eid 2014			0.94	0.41	1.48	
							Sullivan 2017			0.27	0.03	0.51	
Pooled ES			*0.43*	*-0.31*	*1.17*		*Pooled ES*			*0.56*	*-0.09*	*1.21*	
**Prophylactic midurethral sling/ Occult urinary incontinence**	3	231				0 %		1	337				
Fuentes 2011			0.78	0.22	1.33		Wei 2012			0.38	0.24	0.52	
Schierlitz 2014			1.02	0.12	1.92								

© 2022 Karjalainen T et al. *JAMA Network Open.*

		Non-placebo							Placebo				
**Intervention/ Condition**	**N Studies**	**N Observations**	**Effect size**	**95%CI**		**I²**	**Study**	**N Studies**	**N Observations**	**Effect size**	**95%CI**		**I²**
van der Ploeg 2016			0.99	0.69	1.29								
Pooled ES			*0.95*	*0.69*	*1.20*		*Pooled ES*			*0.38*	*0.24*	*0.52*	
**Transplantation of fetal cells/ Parkinson's disease**	1	7						1	40				
Spencer 1992			0.23	-1.06	1.51		Freed 2001			0.27	-0.34	0.88	
Pooled ES			*0.23*	*-1.06*	*1.51*		*Pooled ES*			*0.27*	*-0.34*	*0.88*	
**Scalpel debridement/ Plantar callus**	1	65						2	118				23 %
Siddle 2013			0.03	-0.46	0.51		Davys 2005			0.08	-0.55	0.71	
							Landorf 2013			0.53	0.08	0.97	
Pooled ES			*0.03*	*-0.46*	*0.51*		*Pooled ES*			*0.36*	*-0.06*	*0.78*	
**Arthroscopi subacromial decompression/ Rotator cuff disease**	8	805				79 %		2	278				0 %
Beard 2018			0.24	-0.13	0.61		Beard (Q3) 2018			0.13	-0.24	0.49	
Brox 1993			-0.90	-1.35	-0.45		Paavola 2018			0.16	-0.21	0.53	
Farfaras 2016			0.61	0.06	1.16								
Haahr 2005			-0.16	-0.58	0.27								
Ketola 2009			0.56	0.19	0.94								
Paavola 2018			0.36	-0.01	0.72								
Peters 1997			0.06	-0.44	0.56								
Rahme 1998			0.36	-0.03	0.76								
Pooled ES			*0.14*	*-0.18*	*0.47*		*Pooled ES*			*0.14*	*-0.12*	*0.40*	
**Autologous olfactory transplant/ Spinal cord injury**	1	28						1	12				
Chen 2014 a			0.75	-0.68	2.19		Wang 2016			0.17	-0.94	1.28	
Pooled ES			*0.75*	*-0.68*	*2.19*		*Pooled ES*			*0.17*	*-0.94*	*1.28*	
**Vertebroplasty/ Vertebral compression fracture**	7	719				91 %		5	561				7 %

© 2022 Karjalainen T et al. *JAMA Network Open.*

		Non-placebo							Placebo				
**Intervention/ Condition**	**N Studies**	**N Observations**	**Effect size**	**95%CI**		**I²**	**Study**	**N Studies**	**N Observations**	**Effect size**	**95%CI**		**I²**
Farrokhi 2011			2.08	1.55	2.62		Firanescu 2018			0.10	-0.20	0.39	
Klazen 2010			1.21	0.90	1.52		Hansen 2016			-0.16	-0.73	0.41	
Rousing 2010			0.18	-0.39	0.75		Kallmes 2009			0.15	-0.19	0.50	
Voormoolen 2006			0.46	-0.21	1.12		Buchbinder 2009			0.20	-0.26	0.65	
Yang 2016			2.28	1.79	2.76		Clark 2016			0.49	0.11	0.86	
Blasco 2012			0.48	0.12	0.83								
Chen a 2014			1.64	1.16	2.11								
Pooled ES			*1.20*	*0.63*	*1.77*		*Pooled ES*			*0.18*	*0.01*	*0.36*	
**Overall**	**41**	**4685**	***0.48***	***0.31***	***0.65***	**89 %**	**Overall**	**32**	**3193**	***0.39***	***0.26***	***0.53***	**76 %**
Sensitivity subset			***0.53***	***0.36***	***0.70***					***0.42***	***0.27***	***0.57***	

[a] GastroEsophageal Reflux Disease

© 2022 Karjalainen T et al. *JAMA Network Open.*

eAppendix 1. Search Strategies
(All searches performed through Ovid)

Trials with placebo surgery controls (21th November, 2018)

Updated search from Wartolowska K, Judge A, Hopewell S, et al. Use of placebo controls in the evaluation of surgery: systematic review. *BMJ* 2014;348:g3253. doi:10.1136/bmj.g3253.

MEDLINE

1. Clinical trial/
2. Randomized controlled trial/
3. Randomization/
4. Rct.tw.
5. random allocation.tw.
6. Randomly allocated.tw.
7. Allocated randomly.tw.
8. Randomized Controlled Trials as Topic/
9. randomized controlled trial/
10. Double Blind Method/
11. Single Blind Method/
12. clinical trial/
13. controlled clinical trial.pt.
14. randomized controlled trial.pt.
15. clinical trial.pt.
16. exp Clinical Trials as topic/
17. or/1-16
18. PLACEBOS/
19. placebo$.tw.
20. sham.tw.
21. immitation.tw.
22. placebo effect$.tw.
23. or/18-22
24. surgery.tw.
25. surgical.tw.
26. arthroscopy.tw.
27. endoscopy.tw.
28. transplantation.tw.
29. $scopy.tw.
30. $scopic.tw.
31. laparoscopy.tw.
32. Meta-Analysis as Topic/
33. meta analy$.tw.
34. metaanaly$.tw.
35. Review/
36. Comment/
37. Letter/
38. Editorial/
39. animal/
40. dose$.tw.
41. pre$medication.tw.
42. an$esthesia.tw.
43. an$esthetic$.tw.
44. antibiotic$.tw.
45. steroid$.tw.
46. prophylaxis.tw.
47. prevention.tw.
48. preoperative.tw.
49. preanaesthetic$.tw.
50. pre$emptive.tw.
51. pre-operative.tw.
52. post-operative.tw.
53. postoperative.tw.
54. post$surgery.tw.
55. (analgesic adj trial).tw.
56. oral$.tw.
57. acupuncture.tw.
58. acupressure.tw.
59. scar.tw.
60. infection.tw.
61. dental.tw.
62. post$surgical.tw.
63. pre$surgical.tw.
64. case report.tw.
65. case study.tw.
66. pacing.tw.
67. stimulation.tw.
68. growth factor$.tw.
69. hormon$.tw.
70. or/24-31
71. or/32-69
72. 17 and 23
73. 72 and 70
74. 73 not 71

EMBASE

1. Clinical trial/
2. Randomized controlled trial/
3. Randomization/
4. Single blind procedure/
5. Double blind procedure/

24. endoscopy.tw.
25. $scopy.tw.
26. $scopic.tw.
27. laparoscopy.tw.
28. transplantation.tw.

47. an$esthetic$.tw.
48. steroid$.tw.
49. peri$operative.tw.
50. pre$emptive.tw.
51. preanesthetic$.tw.

© 2022 Karjalainen T et al. *JAMA Network Open.*

6. Crossover procedure/
7. Randomi?ed controlled trial$.tw.
8. Rct.tw.
9. random allocation.tw.
10. Randomly allocated.tw.
11. Allocated randomly.tw.
12. (allocated adj2 random).tw.
13. Single blind$.tw.
14. Single blind$.tw.
15. or/1-14
16. Placebo$.tw.
17. placebo effect$.tw.
18. sham.tw.
19. placebo.tw.
20. or/16-19
21. surgery.tw.
22. surgical.tw.
23. arthroscopy.tw.
29. or/21-28 30.
30. letter/
31. Review/
32. animal/
33. editorial/
34. ((meta adj analy$) or metaanalys$).tw.
35. (analgesic adj trial).tw.
36. meta$analysis.tw.
37. dose$.tw.
38. oral$.tw.
39. orally.tw.
40. dental.tw.
41. pre$medication.tw.
42. pre$surgical.tw.
43. post$surgical.tw.
44. pre$surgery.tw.
45. post$surgery.tw.
46. antibiotic$.tw.
52. post$operative.tw.
53. prophylaxis.tw.
54. prevention.tw.
55. acupuncture.tw.
56. accupressure.tw.
57. scar$.tw.
58. infection$.tw.
59. acupressure.tw.
60. pre$operative.tw.
61. growth factor$.tw.
62. pacing.tw.
63. stimulation.tw.
64. hormon$.tw.
65. case report$.tw.
66. case study.tw.
67. or/30-66
68. 15 and 20
69. 68 and 29
70. 69 not 67

CENTRAL
1. (placebo OR placebo effect OR sham OR imitation):ti,ab,kw
2. (surgery OR surgical OR laparoscopy OR endoscopy OR arthroscopy OR transplantation OR scopy):ti,ab,kw
3. (clinical trial OR randomised clinical trail OR RCT OR randomised controlled trial OR randomisation):ti,ab,kw
4. not (drug OR dental OR oral OR infection OR steroids OR hormones OR growth factor OR prophylaxis OR anaesthesia OR pre-surgical OR post-surgical OR pre-emptive OR post-operative OR preoperative OR antibiotics OR acupuncture OR acupressure OR scar OR infection OR prevention):ti,ab,kw
5. not (review OR animal OR stimulation):ti,ab,kw
6. #1 AND #2 AND #3 AND #4 AND #5
7. In Trials

Ablation or excision for endometriosis (17th March, 2019)

Updated search from Duffy JM, Arambage K, Correa FJ, et al. Laparoscopic surgery for endometriosis. *Cochrane Database Syst Rev* 2014;(4):CD011031. doi:10.1002/14651858.CD011031.

MEDLINE

1. controlled clinical trial.pt.
2. randomized.ab.
3. randomised.ab.
4. placebo.tw.
5. clinical trials as topic.sh.
6. randomly.ab.
7. trial.ti.
8. (crossover or cross-over or cross over).tw.
18. exp diathermy/
19. diathermy.tw.
20. LUNA.tw.
21. presacral neurectom$.tw.
22. laser$.tw.
23. plasmajet.tw.
24. plasma jet.tw.
25. randomized controlled trial.pt.
35. microsurg$.tw.
36. uterine nerve ablation$.tw.
37. uterosacral nerve ablation.tw.
38. minimally invasive.tw.
39. (ablation or ablative).tw.
40. or/13-39
41. exp endometriosis/
42. exp infertility/

© 2022 Karjalainen T et al. *JAMA Network Open.*

9. or/1-9
10. exp animals/ not humans.sh.
11. 10 NOT 11
12. exp Laparoscopy/
13. Laparoscop$.tw.
14. celioscop$.tw.
15. peritoneoscop$.tw.
16. exp minimally invasive surgery/
17. exp laser/
26. microlaparoscop$.tw.
27. minilaparoscop$.tw.
28. exp robotics/
29. exp computer assisted surgery/
30. Computer-Assisted Surg$.tw.
31. da vinci.tw.
32. (keyhole adj3 surg$).tw.
33. Robot$.tw.
34. remote surg$.tw.
43. endometrio$.tw.
44. dyschezia.tw.
45. dyspareunia.tw.
46. exp infertility/
47. or/41-46
48. AND/12,40,47
49. limit 48 to yr=”2013-current

EMBASE

1. Clinical Trial/
2. Randomized Controlled Trial/
3. exp randomization/
4. Single Blind Procedure/
5. Double Blind Procedure/
6. Crossover Procedure/
7. Placebo/
8. Randomi?ed controlled trial$.tw.
9. Rct.tw.
10. random allocation.tw.
11. randomly allocated.tw.
12. allocated randomly.tw.
13. (allocated adj2 random).tw.
14. Single blind$.tw.
15. Double blind$.tw.
16. ((treble or triple) adj blind$).tw.
17. placebo$.tw.
18. prospective study/
19. or/1-18
20. case study/
21. case report.tw.
22. abstract report/ or letter/
23. or/20-22
24. 19 NOT 23
25. exp Laparoscopy/
26. Laparoscop$.tw.
27. celioscop$.tw.
28. peritoneoscop$.tw.
29. exp minimally invasive surgery/
30. exp laser/
31. exp diathermy/
32. diathermy.tw.
33. LUNA.tw.
34. presacral neurectom$.tw.
35. laser$.tw.
36. plasmajet.tw.
37. plasma jet.tw.
38. microlaparoscop$.tw.
39. minilaparoscop$.tw.
40. exp robotics/
41. exp computer assisted surgery/
42. Computer-Assisted Surg$.tw.
43. da vinci.tw.
44. (keyhole adj3 surg$).tw.
45. Robot$.tw.
46. remote surg$.tw.
47. microsurg$.tw.
48. uterine nerve ablation$.tw.
49. uterosacral nerve ablation.tw.
50. minimally invasive.tw.
51. (ablation or ablative).tw.
52. exp hand assisted laparoscopy/
53. or/25-53
54. exp endometriosis/
55. exp infertility/
56. endometrio$.tw.
57. dyschezia.tw.
58. dyspareunia.tw.
59. or/54-58
60. AND/24,53,59
61. limit 60 to yr=” 2013-current”

CENTRAL

1 exp Laparoscopy/
2 Laparoscop$.ti,ab,sh.
3 celioscop$.tw.
4 peritoneoscop$.tw.
5 exp Surgical Procedures, Minimally Invasive/
6 Lasers/
7 exp Diathermy/
8 LUNA
9 presacral neurectom*
13 plasmajet.tw.
14 plasma jet.tw.
15 excision.tw.
16 microlaparoscop$.tw.
17 minilaparoscop$.tw.
18 exp Robotics/
19 exp Surgery, Computer-Assisted/
20 Computer-Assisted Surg$.tw.
21 da vinci.tw.
25 microsurg$.tw.
26 minimally invasive.tw.
27 (ablation or ablative).tw.
28 or/1-27
29 exp Endometriosis/
30 endometrio$.tw.
31 dyschezia.tw.
32 dyspareunia.tw.
33 infertility:kw

© 2022 Karjalainen T et al. *JAMA Network Open.*

10 (minimal$ adj5 surg$).tw.
11 laser$.tw.
12 diathermy.tw.
22 (keyhole near3 surg$).tw.
23 Robot$.tw.
24 remote surg$.tw.
34 MeSH term infertility explode all trees
35 #29 or #30 or #31 or 32 #or 33
34 28 and 35

Adhesiolysis for chronic abdominal pain (8th March, 2019)

Updated search from van den Beukel BA, de Ree R, van Leuven S,et al. Surgical treatment of adhesion-related chronic abdominal and pelvic pain after gynaecological and general surgery: a systematic review and meta-analysis. *Hum Reprod Update* 2017;23:276-288. doi:10.1093/humupd/dmx004.

MEDLINE

1. randomized controlled trial.pt.
2. controlled clinical trial.pt.
3. randomized.ab.
4. randomised.ab.
5. placebo.tw.
6. clinical trials as topic.sh.
7. randomly.ab.
8. trial.ti.
9. groups.tiab.
10. OR/1-9
11. exp animals/ not humans.sh.
12. 10 NOT 11
13. exp abdominal pain/
14. exp chronic pain/
15. exp Tissue Adhesions/
16. Adhesion$.tw.
17. adhesi*.tiab.
18. OR/12-17
19. exp laparotomy
20. exp laparoscopy
21. laparoscop*.ti,ab.
22. laparotomy.ti,ab.
23. adhesiolysis.ti,ab.
24. ((abdomen or abdominal or abdomino*) and surgery).ti,ab.
25. OR/18-24
26. AND/11,18,25
27. limit 26 to yr=”2016-Current”

EMBASE

1. Clinical Trial/
2. Randomized Controlled Trial/
3. exp randomization/
4. Single Blind Procedure/
5. Double Blind Procedure/
6. Crossover Procedure/
7. Placebo/
8. Randomi?ed controlled trial$.tw.
9. Rct.tw.
10. random allocation.tw.
11. randomly allocated.tw.
12. allocated randomly.tw.
13. (allocated adj2 random).tw.
14. Single blind$.tw.
15. Double blind$.tw.
16. ((treble or triple) adj blind$).ti.
17. placebo$.tw.
18. prospective study/
19. OR/1-18
20. exp abdominal pain/
21. exp chronic pain/
22. exp Tissue Adhesions/
23. Adhesion$.ti.
24. adhesi$.ti,ab.
25. OR/20-24
26. exp laparoscopy/
27. exp laparotomy/
28. laparoscop$.ti,ab.
29. laparotomy.ti,ab.
30. adhesiolysis.ti,ab.
31. (abdomen.ti,ab. or abdominal.ti,ab. or abdomino$.ti,ab.) AND surgery.ti,ab.
32. OR/26-31
33. AND/19,25,32
34. limit 33 to yr=”2016-current”

CENTRAL

1. (abdominal pain)
2. MeSH descriptor [chronic pain)] explode all trees
3. (Adhesion)
4. #1 OR #2 OR #3
5. MeSH descriptor [laparoscopy] explode all trees
6. MeSH descriptor [laparotomy] explode all trees
7. laparoscop$
8. adhesiolysis
9. #5 OR #6 OR #7 OR #8
10. #4 AND #9

© 2022 Karjalainen T et al. *JAMA Network Open.*

Brain stimulation for cervical dystonia (15th April, 2019)

MEDLINE

1. randomized controlled trial.pt.
2. controlled clinical trial.pt.
3. randomized.ab.
4. randomised.ab.
5. placebo.tw.
6. clinical trials as topic.sh.
7. randomly.ab.
8. trial.ti.
9. (crossover or cross-over or cross over).tw.
10. or/1-9
11. exp animals/ not humans.sh.
12. 10 NOT 11
13. cervical dystonia
14. Spasmodic Torticollis
15. focal dystonia
16. laterocollis or anterocollis or retrocollis):tw
17. OR/13-16
18. surgical procedures, operative/
19. (surg* or surgical* or operat*).ti,ab
20. deep brain stimulation.ti,ab
21. OR/18-20
22. and/12,17,21

EMBASE

1. Clinical Trial/
2. Randomized Controlled Trial/
3. exp randomization/
4. Single Blind Procedure/
5. Double Blind Procedure/
6. Crossover Procedure/
7. Placebo/
8. Randomi?ed controlled trial$.tw.
9. Rct.tw.
10. random allocation.tw.
11. randomly allocated.tw.
12. allocated randomly.tw.
13. (allocated adj2 random).tw.
14. Single blind$.tw.
15. Double blind$.tw.
16. ((treble or triple) adj blind$).tw.
17. placebo$.tw.
18. prospective study/
19. or/1-18
20. case study/
21. case report.tw.
22. abstract report/ or letter/
23. or/20-22
24. 19 NOT 23
25. cervical dystonia/
26. spasmotic torticollis.tw.
27. dystonia.ti,ab.
28. laterocollis or anterocollis or retrocollis).tw.
29. OR/25-28
30. surgical procedures, operative/
31. (surg* or surgical* or operat*).ti,ab
32. Deep brain stimulation.ti,ab.
33. or/30-32
34. and/24,29,33

CENTRAL

1. MeSH Term: [Torticollis] explode all trees
2. dystonia:kw
3. #1 OR #2
4. MeSH Term [surgical procedures, operative] explode all trees
5. (surg* or surgical* or operat*):ti,ab
6. (deep brain stimulation):kw
7. #4 or #5 or #6
8. #3 and #7
9. limit to trials

Deep brain stimulation for tardive dystonia (7th May, 2019)

MEDLINE

1. randomized controlled trial.pt.
2. controlled clinical trial.pt.
3. randomized.ab.
4. randomised.ab.
5. placebo.tw.
6. clinical trials as topic.sh.
8. trial.ti.
9. (crossover or cross-over or cross over).tw.
10. or/1-9
11. exp animals/ not humans.sh.
12. 10 NOT 11
13. dystonia/
15. OR/13-14
16. surgical procedures, operative/
17. (surg* or surgical* or operat*).ti,ab
18. deep brain stimulation.ti,ab
19. OR/16-18
20. AND/12,15,19

© 2022 Karjalainen T et al. *JAMA Network Open.*

7. randomly.ab.

14. tardive dystonia.ti,ab.

EMBASE

1. Clinical Trial/
2. Randomized Controlled Trial/
3. exp randomization/
4. Single Blind Procedure/
5. Double Blind Procedure/
6. Crossover Procedure/
7. Placebo/
8. Randomi?ed controlled trial$.tw.
9. Rct.tw.
10. random allocation.tw.
11. randomly allocated.tw.
12. allocated randomly.tw.
13. (allocated adj2 random).tw.
14. Single blind$.tw.
15. Double blind$.tw.
16. ((treble or triple) adj blind$).tw.
17. placebo$.tw.
18. prospective study/
19. or/1-18
20. case study/
21. case report.tw.
22. abstract report/ or letter/
23. or/20-22
24. 19 NOT 23
25. Exp dystonia/
26. tardive dystonia.ti,ab.
27. OR/25-26
28. surgical procedures, operative/
29. (surg* or surgical* or operat*).ti,ab
30. Deep brain stimulation.ti,ab.
31. or/28-30
32. and/24,27,31

CENTRAL

1. dystonia:kw
2. (tardive dystonia):kw
3. (tarvide dyskinesia):kw
4. #1 OR #2 OR #3
5. MeSH Term [surgical procedures, operative] explode all trees
6. (surg* or surgical* or operat*):ti,ab
7. (deep brain stimulation):kw
8. #5 or #6 or #7
9. #4 and #8
10. limit to trials

Scalpel debridement for plantar callus (13th March, 2019)

MEDLINE

1. randomized controlled trial.pt.
2. controlled clinical trial.pt.
3. randomized.ab.
4. randomised.ab.
5. placebo.tw.
6. clinical trials as topic.sh.
7. randomly.ab.
8. trial.ti.
9. (crossover or cross-over or cross over).tw.
10. or/1-9
11. exp animals/ not humans.sh.
12. 10 NOT 11
13. Callosities/
14. callosities.mp.
15. callus.mp.
16. OR/13-15
17. surgical procedures, operative/
18. (surg* or surgical* or operat*).ti,ab.
19. debridement*.ti,ab.
20. OR/17-19
21. AND/12,16,20

EMBASE

1. Clinical Trial/
2. Randomized Controlled Trial/
3. exp randomization/
4. Single Blind Procedure/

13. (allocated adj2 random).tw.
14. Single blind$.tw.
15. Double blind$.tw.
16. ((treble or triple) adj blind$).tw.

24. 19 NOT 23
25. Callosities/
26. callosities.mp.
27. callus.mp.

© 2022 Karjalainen T et al. *JAMA Network Open.*

5. Double Blind Procedure/
6. Crossover Procedure/
7. Placebo/
8. Randomi?ed controlled trial$.tw.
9. Rct.tw.
10. random allocation.tw.
11. randomly allocated.tw.
12. allocated randomly.tw.
17. placebo$.tw.
18. prospective study/
19. or/1-18
20. case study/
21. case report.tw.
22. abstract report/ or letter/
23. or/20-22
28. OR/25-27
29. Exp surgical procedures, operative/
30. (surg$ or surgical$ or operat$).ti,ab.
31. debridement*.ti,ab.
32. OR/29-31
33. AND/24,28,32

CENTRAL

1. MeSH description [Callosities] explode all trees
2. callosit*
3. callus
4. #1 or #2 or #3
5. surgical procedures, operative explode all trees
6. surg* or surgical* or operat*:ti,ab
7. debridement:kw,ti,ab
8. #5 or #6 or #7
9. #4 and #8

Transplantation of human retinal pigment epithelial cells; Transplantation of fetal cells; Transplantation of AAV2 neurturin gene for Parkinson's disease (13th March, 2019)

MEDLINE

1. randomized controlled trial.pt.
2. controlled clinical trial.pt.
3. randomized.ab.
4. randomised.ab.
5. placebo.tw.
6. clinical trials as topic.sh.
7. randomly.ab.
8. trial.ti.
9. (crossover or cross-over or cross over).tw.
10. or/1-9
11. exp animals/ not humans.sh.
12. 10 NOT 11
13. Parkinson's disease/
14. Parkinson's syndrome
15. Parkinson*.ti,ab
16. OR/13-15
17. surgical procedures, operative/
18. (surg* or surgical* or operat*).ti,ab
19. cell adj2 delivery.ti,ab.
20. gene adj delivery.ti,ab.
21. OR/17-20
22. and/12,16,21

EMBASE

1. Clinical Trial/
2. Randomized Controlled Trial/
3. exp randomization/
4. Single Blind Procedure/
5. Double Blind Procedure/
6. Crossover Procedure/
7. Placebo/
8. Randomi?ed controlled trial$.tw.
9. Rct.tw.
10. random allocation.tw.
11. randomly allocated.tw.
12. allocated randomly.tw.
13. (allocated adj2 random).tw.
14. Single blind$.tw.
15. Double blind$.tw.
16. ((treble or triple) adj blind$).tw.
17. placebo$.tw.
18. prospective study/
19. or/1-18
20. case study/
21. case report.tw.
22. abstract report/ or letter/
23. or/20-22
24. 19 NOT 23
25. Parkinson's disease/
26. Parkinson's syndrome
27. Parkinson*.ti,ab
28. OR/25-27
29. surgical procedures, operative/
30. (surg* or surgical* or operat*).ti,ab
31. cell adj2 delivery.ti,ab.
32. gene adj delivery.ti,ab.
33. or/29-32
34. and/24,28,33

CENTRAL

© 2022 Karjalainen T et al. *JAMA Network Open.*

1. MeSH Term: [Parkinson's disease] explode all trees
2. Parkinson*.ti,ab
3. #1 OR #2
4. MeSH Term [surgical procedures, operative] explode all trees
5. (surg* or surgical* or operat*):ti,ab
6. (cell adj2 delivery):ti,ab.
7. (gene adj delivery):ti,ab.
8. #4 or #5 or #6 or #7
9. #3 and #8

Endolymphatic shunt for Meniere (12th March, 2019)

MEDLINE

1. randomized controlled trial.pt.
2. controlled clinical trial.pt.
3. randomized.ab.
4. randomised.ab.
5. placebo.tw.
6. clinical trials as topic.sh.
7. randomly.ab.
8. trial.ti.
9. (crossover or cross-over or cross over).tw.
10. or/1-9
11. exp animals/ not humans.sh.
12. 10 NOT 11
13. shunt.tw.
14. endolymphatic sac adj3 surgery
15. ((endolymphatic or sac) and shunt).ti,ab.
16. (endolymphatic and (surg* or decompress* or drainage)).ti,ab.
17. OR/13-15
18. endolymphatic hydrops.mp.
19. meniere disease/
20. vertigo.mp.
21. labyrinth or aural or endolymphatic adj3 syndrome or vertigo or hydrops
22. OR/18-21

EMBASE

1. Clinical Trial/
2. Randomized Controlled Trial/
3. exp randomization/
4. Single Blind Procedure/
5. Double Blind Procedure/
6. Crossover Procedure/
7. Placebo/
8. Randomi?ed controlled trial$.tw.
9. Rct.tw.
10. random allocation.tw.
11. randomly allocated.tw.
12. allocated randomly.tw.
13. (allocated adj2 random).tw.
14. Single blind$.tw.
15. Double blind$.tw.
16. ((treble or triple) adj blind$).tw.
17. placebo$.tw.
18. prospective study/
19. or/1-18
20. case study/
21. case report.tw.
22. abstract report/ or letter/
23. shunt.tw.
24. endolymphatic sac adj3 surgery
25. ((endolymphatic or sac) and shunt).ti,ab.
26. (endolymphatic and (surg$ or decompress$ or drainage)).ti,ab.
27. OR/23-26
28. endolymphatic hydrops.mp.
29. meniere disease/
30. vertigo.mp.
31. labyrinth or aural or endolymphatic adj3 syndrome or vertigo or hydrops
32. OR/28-31

CENTRAL

1. surg* or decompression or drainage or shunt or operat* or surgical*
2. endolymphatic
3. #1 AND #2

Extensor carpi radialis brevis release for tennis elbow (14th March, 2019)
Updated search from Buchbinder R, Green S, Bell S,et al. Surgery for lateral elbow pain. *Cochrane Database Syst Rev* 2002;(1):CD003525. doi: 10.1002/14651858.CD003525. Update in: *Cochrane Database Syst Rev* 2011;(3):CD003525.

MEDLINE

© 2022 Karjalainen T et al. *JAMA Network Open.*

1. randomized controlled trial.pt.
2. controlled clinical trial.pt.
3. randomized.ab.
4. randomised.ab.
5. placebo.tw.
6. clinical trials as topic.sh.
7. randomly.ab.
8. trial.ti.
9. (crossover or cross-over or cross over).tw.
10. or/1-9
11. exp animals/ not humans.sh.
12. 10 NOT 11
13. exp Tennis Elbow/
14. exp Tendinopathy/
15. exp Tendon Injuries/
16. exp Elbow Joint/
17. exp Pain/
18. 16 and 17
19. tennis elbow.tw.
20. (Tendinitis or Tendinosis or Tendonitis).tw.
21. (pain$ and lateral elbow).tw.
22. epicondylitis.tw.
23. common extensor origin.tw.
24. epicondylalgia.tw.
25. or/13-15,18-24
26. exp Surgery/
27. (surgery$ or surgeries or surgical or operat$).tw.
28. (tenotomy or tendon release or ECRB release or extensor carpi radialis brevis release).ti,ab.
29. or/26-28
30. AND/12,25,29

EMBASE

1. Clinical Trial/
2. Randomized Controlled Trial/
3. exp randomization/
4. Single Blind Procedure/
5. Double Blind Procedure/
6. Crossover Procedure/
7. Placebo/
8. Randomi?ed controlled trial$.tw.
9. Rct.tw.
10. random allocation.tw.
11. randomly allocated.tw.
12. allocated randomly.tw.
13. (allocated adj2 random).tw.
14. Single blind$.tw.
15. Double blind$.tw.
16. ((treble or triple) adj blind$).tw.
17. placebo$.tw.
18. prospective study/
19. or/1-18
20. case study/
21. case report.tw.
22. abstract report/ or letter/
23. or/20-22
24. 19 NOT 23
25. exp Tennis Elbow/
26. exp Tendinopathy/
27. exp Tendon Injuries/
28. exp Elbow Joint/
29. exp Pain/
30. 28 and 29
31. tennis elbow.tw.
32. (Tendinitis or Tendinosis or Tendonitis).tw.
33. (pain$ and lateral elbow).tw.
34. epicondylitis.tw.
35. common extensor origin.tw.
36. epicondylalgia.tw.
37. or/25-27,30-36
38. exp Surgery/
39. (surgery$ or surgeries or surgical or operat$).ti,ab.
40. (tenotomy or tendon release or ECRB release or extensor carpi radialis brevis release).ti,ab.
41. or/26-28
42. AND/12,25,29

CENTRAL

1. MeSH descriptor: [Tennis Elbow] explode all trees
2. MeSH descriptor: [Elbow Tendinopathy] explode all trees
3. MeSH descriptor: [Tendon Injuries] explode all trees
4. MeSH descriptor: [Tendon Injuries] explode all trees
5. MeSH descriptor: [Pain] explode all trees
6. #4 and #5
7. tennis elbow:ti,ab
8. (Tendinitis or Tendinosis or Tendonitis):ti,ab
9. (pain* and “lateral elbow”):ti,ab
10. epicondylitis:ti,ab
11. “common extensor origin”:ti,ab
12. epicondylalgia:ti,ab
13. (#1 OR #2 OR #3 OR #6 OR #7 OR #8 OR #9 OR #10 OR #11 OR #12)
14. MeSH descriptor: [Surgical Procedures, Operative] explode all trees
15. (surgery* or surgeries or surgical or operat*):ti,ab
16. (tenotomy or tendon release or ECRB release or extensor carpi radialis brevis release):ti,ab
17. #14 or #15 or #16
18. #13 and #17

Left internal mammary artery ligation for coronary heart disease (8th March, 2019)

© 2022 Karjalainen T et al. *JAMA Network Open.*

MEDLINE and EMBASE

1. internal mammary artery ligation)
2. (internal-mammary-artery ligation)
3. division adj3 (internal mammary arter*)
4. OR/1-3

CENTRAL

1. internal mammary artery ligation
2. internal-mammary-artery ligation
3. division
4. internal mammary arter*
5. #3 AND #4
6. #1 OR #2 OR #5

Low flow shunt for Alzheimer's disease (28th July, 2020)

MEDLINE

1. randomized controlled trial.pt.
2. controlled clinical trial.pt.
3. randomized.ab.
4. randomised.ab.
5. placebo.tw.
6. clinical trials as topic.sh.
7. randomly.ab.
8. trial.ti.
9. (crossover or cross-over or cross over).tw.
10. or/1-9
11. exp animals/ not humans.sh.
12. 10 NOT 11
13. Alzheimer*.ti,ab.
14. Alzheimer Disease/
15. AD.ti,ab.
16. "cognit* impair*".ti,ab.
17. OR/13-16
18. surgical procedures, operative/
19. (surg* or surgical* or operat*).ti,ab
20. cognishunt
21. (ventriculoperitoneal shunt)
22. OR/18-21
23. and/12,17,22

EMBASE

1. Clinical Trial/
2. Randomized Controlled Trial/
3. exp randomization/
4. Single Blind Procedure/
5. Double Blind Procedure/
6. Crossover Procedure/
7. Placebo/
8. Randomi?ed controlled trial$.tw.
9. Rct.tw.
10. random allocation.tw.
11. randomly allocated.tw.
12. allocated randomly.tw.
13. (allocated adj2 random).tw.
14. Single blind$.tw.
15. Double blind$.tw.
16. ((treble or triple) adj blind$).tw.
17. placebo$.tw.
18. prospective study/
19. or/1-18
20. case study/
21. case report.tw.
22. abstract report/ or letter/
23. or/20-22
24. 19 NOT 23
25. Alzheimer*.ti,ab.
26. Alzheimer Disease/
27. AD.ti,ab.
28. "cognit* impair*".ti,ab.
29. OR/25-28
30. surgical procedures, operative/
31. (surg* or surgical* or operat*).ti,ab
32. (ventriculoperitoneal shunt).ti,ab
33. Cognishunt
34. or/30-33
35. and/24,29,34

CENTRAL

1. MeSH Term: Alzheimer explode all trees
2. MeSH Term [surgical procedures, operative] explode all trees
3. (surg* or surgical* or operat*):ti,ab
4. (ventriculoperitoneal shunt):kw
5. cognishunt:kw
6. #2 or #3 or #4 or #5
7. #3 and #8
8. limit to trials

© 2022 Karjalainen T et al. *JAMA Network Open.*

Endoscopic gastric plication; Transoral outlet suture; Vagal nerve stimulation for Obesity (13th March, 2019)

Updated from Colquitt JL, Pickett K, Loveman E, et al. Surgery for weight loss in adults. *Cochrane Database Syst Rev* 2014;(8):CD003641. doi:10.1002/14651858.CD003641.pub4.

MEDLINE

1. randomized controlled trial.pt.
2. controlled clinical trial.pt.
3. randomized.ab.
4. randomised.ab.
5. placebo.tw.
6. clinical trials as topic.sh.
7. randomly.ab.
8. trial.ti.
9. (crossover or cross-over or cross over).tw.
10. or/1-9
11. exp animals/ not humans.sh.
12. 10 NOT 11
13. exp obesity/
14. Overweight/
15. over?weight.ti,ab.
16. over weight.ti,ab.
17. overeating.ti,ab.
18. over?eating.ti,ab.
19. exp Weight Loss/
20. weight loss.ti,ab.
21. weight reduc$.ti,ab.
22. or/13-21
23. bariatric surg$.ti,ab.
24. exp bariatric surgery/
25. (surg$ adj5 bariatric).ti,ab.
26. anti?obesity surg$.ti,ab.
27. antiobesity surg$.ti,ab.
28. (obesity adj5 surgery).ti,ab.
29. (obesity adj5 surgical).ti,ab.
30. (gastroplasty or gastro?gastostomy or ”gastric bypass“ or ”gastric surgery“ or ”restrictive surgery“).ti,ab.
31. exp gastric bypass/
32. gastroplasty/
33. ((gastric plication) or (vagal nerve stimulation) OR (vagal nerve block)).ti,ab.
34. stomach stapl$.ti,ab.
35. obesity/su
36. exp Obesity, Morbid/su [Surgery]
37. OR/23-36
38. AND/12,22,37

EMBASE

1. Clinical Trial/
2. Randomized Controlled Trial/
3. exp randomization/
4. Single Blind Procedure/
5. Double Blind Procedure/
6. Crossover Procedure/
7. Placebo/
8. Randomi?ed controlled trial$.tw.
9. Rct.tw.
10. random allocation.tw.
11. randomly allocated.tw.
12. allocated randomly.tw.
13. (allocated adj2 random).tw.
14. Single blind$.tw.
15. Double blind$.tw.
16. ((treble or triple) adj blind$).tw.
17. placebo$.tw.
18. prospective study/
19. or/1-18
20. case study/
21. case report.tw.
22. abstract report/ or letter/
23. or/20-22
24. 19 NOT 23
25. exp OBESITY/ or exp MORBID OBESITY/
26. over?weight.ti,ab.
27. over weight.ti,ab.
28. overeating.ti,ab.
29. over?eating.ti,ab.
30. exp Weight Reduction/
31. (weight adj1 los*).ti,ab.
32. (weight adj1 loos*).ti,ab.
33. weightloss.ti,ab.
34. weight?loss.ti,ab.
35. (weight adj3 reduc*).ti,ab.
36. weight?reduc*.ti,ab.
37. or/25-36
38. bariatric surg*.ti,ab.
39. exp Bariatric Surgery/
40. (surg* adj5 bariatric).ti,ab.
41. (anti?obesity adj3 surg*).ti,ab.
42. (antiobesity adj3 surg*).ti,ab.
43. anti obesity surg*.ti,ab.
44. (obesity adj5 surgery).ti,ab.
45. (obesity adj5 surgical).ti,ab.
46. (gastroplasty or gastrogastrostomy or gastro?gastrostomy or gastroenterostomy or gastric bypass or gastric surgery or
47. restrictive surgery).ti,ab.
48. exp GASTROPLASTY/
49. (“gastric plication” or “vagal nerve stimulation” OR “vagal nerve block”).ti,ab.
50. gastric stapl*.ti,ab.
51. OR/38-50
52. 37 AND 51
53. OBESITY/su [Surgery]
54. Morbid Obesity/su [Surgery]
55. 53 OR 54
56. 37 AND 55
57. 52 OR 56

© 2022 Karjalainen T et al. *JAMA Network Open.*

CENTRAL

#1 MeSH descriptor: [Obesity] explode all trees
#2 MeSH descriptor: [Overweight] this term only
#3 MeSH descriptor: [Weight Loss] explode all trees
#4 (obes* or overweight or ”over weight“)
#5 #1 or #2 or #3 or #4
#6 MeSH descriptor: [Bariatric Surgery] explode all trees
#7 (bariatric near/5 surg*)
#8 (obes* near/5 surg*)
#9 antiobesity or anti-obesity or anti obesity near/5 (surg*))
#10(gastroplasty or gastrogastrostomy or gastro?gastrostomy or gastroenterostomy or ”gastric bypass“ or ”gastric surgery“ or ”restrictive surgery“)
#11 MeSH descriptor: [Gastric Bypass] explode all trees
#12 MeSH descriptor: [Gastroplasty] explode all trees
#13 stomach near/5 stapl*
#14 gastric near/5 stapl*
#15 (gastric plication):ti,ab OR (vagal nerve block):ti,ab OR (vagal nerve stimulation):ti,ab

Autologous olfactory transplant for spinal cord injury (14th March, 2019)

MEDLINE

1. randomized controlled trial.pt.
2. controlled clinical trial.pt.
3. randomized.ab.
4. randomised.ab.
5. placebo.tw.
6. clinical trials as topic.sh.
7. randomly.ab.
8. trial.ti.
9. (crossover or cross-over or cross over).tw.
10. or/1-9
11. exp animals/ not humans.sh.
12. 10 NOT 11
13. exp Spinal Cord Injuries/
14. exp Central Cord Syndrome/
15. (myelopathy adj3 (traumatic or post-traumatic)).ab,ti.
16. ((spine or spinal or vertebrae) adj3 (fracture* or wound* or trauma* or injur* or damag*)).ab,ti.
17. (spinal cord adj3 (contusion or laceration or transaction or trauma or ischemia)).ab,ti.
18. central cord injury syndrome.ab,ti.
19. central spinal cord syndrome.ab,ti.
20. exp Paraplegia/
21. exp Quadriplegia/
22. OR/13-21
23. cell adj3 transplantation
24. Lamina Propria Transplantation
25. transplant*
26. regenerative surgery
27. AND/12,22,26

EMBASE

1. Clinical Trial/
2. Randomized Controlled Trial/
3. exp randomization/
4. Single Blind Procedure/
5. Double Blind Procedure/
6. Crossover Procedure/
7. Placebo/
8. Randomi?ed controlled trial$.tw.
9. Rct.tw.
10. random allocation.tw.
11. randomly allocated.tw.
12. allocated randomly.tw.
15. Double blind$.tw.
16. ((treble or triple) adj blind$).tw.
17. placebo$.tw.
18. prospective study/
19. or/1-18
20. case study/
21. case report.tw.
22. abstract report/ or letter/
23. or/20-22
24. 19 NOT 23
25. exp Spinal Cord Injuries/
26. exp Central Cord Syndrome/
29. (spinal cord adj3 (contusion or laceration or transaction or trauma or ischemia)).ab,ti.
30. central cord injury syndrome.ab,ti.
31. central spinal cord syndrome.ab,ti.
32. exp Paraplegia/
33. exp Quadriplegia/
34. OR/25-33
35. cell adj3 transplantation
36. Lamina Propria/
37. transplant$
38. regenerative surgery
39. OR/35-38

© 2022 Karjalainen T et al. *JAMA Network Open.*

13. (allocated adj2 random).tw.
14. Single blind$.tw.
27. (myelopathy adj3 (traumatic or post-traumatic)).ab,ti.
28. ((spine or spinal or vertebrae) adj3 (fracture$ or wound$ or trauma$ or injur$ or damage$)).ab,ti.
40. AND/24,34,39

CENTRAL

1. MeSH descriptor: [Spinal Cord Injuries] explode all trees
2. MeSH descriptor: [Central Cord Syndrome] explode all trees
3. myelopathy near3 (traumatic or post-traumatic)
4. (spine or spinal or vertebrae) near3 (fracture* or wound* or trauma* or injur* or damag*)
5. (spinal cord) near3 (contusion or laceration or transaction or trauma or ischemia)
6. central cord injury syndrome
7. central spinal cord syndrome
8. paraplegi* or quadriplegi* or tetraplegi*
9. #1 OR #2 OR #3 OR #4 OR #5 OR #6 OR #7 OR #8
10. transplant*:kw
11. cell near3 transplantation
12. regenerative surgery
13. MeSH descriptor: [Mucous Membrane] explode all trees
14. lamina propria transplant*:kw
15. #10 or #11 or #12 #13 or #14
16. #9 and #15 (in trials)

Palatal implants or nasal surgery for sleep apnea (14th March, 2019)

MEDLINE

1. randomized controlled trial.pt.
2. controlled clinical trial.pt.
3. randomized.ab.
4. randomised.ab.
5. placebo.tw.
6. clinical trials as topic.sh.
7. randomly.ab.
8. trial.ti.
9. (crossover or cross-over or cross over).tw.
10. or/1-9
11. exp animals/ not humans.sh.
12. 10 NOT 11
13. palate/
14. palatal OR palate
15. 13 OR 14
16. implant*
17. 15 AND 16
18. septumplasty/
19. nasal adj3 surgery.ti,ab.
20. resection adj4 septum.tw.
21. OR/17-20
22. obstructive sleep apnea/
23. sleep apnea.ti,ab.
24. sleeping disorder.ti,ab
25. OR/22-24
26. AND/12,21,25

EMBASE

1. Clinical Trial/
2. Randomized Controlled Trial/
3. exp randomization/
4. Single Blind Procedure/
5. Double Blind Procedure/
6. Crossover Procedure/
7. Placebo/
8. Randomi?ed controlled trial$.tw.
9. Rct.tw.
10. random allocation.tw.
11. randomly allocated.tw.
14. Single blind$.tw.
15. Double blind$.tw.
16. ((treble or triple) adj blind$).tw.
17. placebo$.tw.
18. prospective study/
19. or/1-18
20. case study/
21. case report.tw.
22. abstract report/ or letter/
23. or/20-22
24. 19 NOT 23
27. palatal OR palate.mp.
28. 25 OR 26
29. implant*.mp.
30. 27 and 28
31. exp septumplasty/
32. nasal surgery/
33. nasal adj3 surgery.ti,ab.
34. resection adj4 septum.tw.
35. OR/29-33
36. exp obstructive sleep apnea/
37. sleep apnea.ti,ab.

© 2022 Karjalainen T et al. *JAMA Network Open.*

12. allocated randomly.tw.
13. (allocated adj2 random).tw.
25. palate/
26.
38. sleeping disorder*.ti,ab
39. OR/35-37
40. AND/24,34,38

CENTRAL

1. MeSH descriptor: [Palate] explode all trees
2. palatal or palate:kw
3. implant*
4. #1 or #2
5. #3 and #4
6. MeSH descriptor: [Nasal Surgical Procedures] explode all trees
7. nasal adj3 surgery:ti,ab
8. septum adj4 resection
9. #5 OR #6 OR #7 OR #8
10. MeSH descriptor: [Sleep Apnea, Obstructive] explode all trees
11. sleep apnea:ti,ab
12. sleep disorder*:kw
13. #10 or #11 or #12
14. #9 and #13

Prophylactic midurethral sling for occult urinary incontinence and autologous fat transplant for urinary incontinence (14th March, 2019)

Updated search from: 1) Kirchin V, Page T, Keegan PE, et al. Urethral injection therapy for urinary incontinence in women. *Cochrane Database Syst Rev* 2012;(2):CD003881. doi: 10.1002/14651858.CD003881.pub3. Update in: *Cochrane Database Syst Rev* 2017;7:CD003881.
2) Maher C, Feiner B, Baessler K, et al. Surgery for women with anterior compartment prolapse. *Cochrane Database Syst Rev*. 201630;11:CD004014. doi: 10.1002/14651858.CD004014.pub6.

MEDLINE

1. randomized controlled trial.pt.
2. controlled clinical trial.pt.
3. randomized.ab.
4. randomised.ab.
5. placebo.tw.
6. clinical trials as topic.sh.
7. randomly.ab.
8. trial.ti.
9. (crossover or cross-over or cross over).tw.
10. or/1-9
11. exp animals/ not humans.sh.
12. 10 NOT 11
13. Urinary Incontinence, Stress/
14. ((stress* or mix* or urg* or urin*) adj3 incontinen$).tw.
15. stress urinary incontinence*.mp.
16. occult urinary incontinence.mp.
17. OR/13-16
18. surgical procedures, operative/
19. (surg* or surgical* or operat*).ti,ab.
20. suburethral sling.mp.
21. abdominal sling.mp.
22. traditional sling procedure$*.tw.
23. suburethral sling procedure.tw.
24. mid$urethral sling.tw.
25. retropubic sling procedure$*.tw.
26. transobturator sling procedure$.tw.
27. TVT-Secur.mp.
28. mini-arc or mini-arc.mp.
29. ajust.mp.
30. needleless.mp.
31. solyx.mp.
32. single$incision sling$.mp.
33. mini$sling.mp.
34. Ophira.mp.
35. Tissue Fixation System.mp.
36. OR/18-35
37. ((urethra* or periurethra* or transurethra*) adj3 (agent* or bulk* or injection* or injectable*)).tw.
38. injection therapy.tw.
39. injectable$.tw.
40. (injectable$ adj2 agent$).tw.
41. (bulk$ adj3 agent$).tw.
42. autologous fat.mp.
43. Peri$urethral injection$.mp.
44. OR/38-44
45. AND/12,17,36 (for the sling, limit 2018 – current)
46. AND/12, 17, 44 (2017-current)

EMBASE

1. Clinical Trial/
2. Randomized Controlled Trial/
21. case report.tw.
22. abstract report/ or letter/
41. ajust.mp.
42. needleless.mp.

© 2022 Karjalainen T et al. *JAMA Network Open.*

3. exp randomization/
4. Single Blind Procedure/
5. Double Blind Procedure/
6. Crossover Procedure/
7. Placebo/
8. Randomi?ed controlled trial$.tw.
9. Rct.tw.
10. random allocation.tw.
11. randomly allocated.tw.
12. allocated randomly.tw.
13. (allocated adj2 random).tw.
14. Single blind$.tw.
15. Double blind$.tw.
16. ((treble or triple) adj blind$).tw.
17. placebo$.tw.
18. prospective study/
19. or/1-18
20. case study/
23. or/20-22
24. 19 NOT 23
25. exp Urinary Incontinence, stress/
26. ((stress$ or mix$ or urg$ or urin$) adj3 incontinen$).tw.
27. stress urinary incontinence*.mp.
28. occult urinary incontinence.mp.
29. OR/25-28
30. exp surgical procedures, operative/
31. (surg* or surgical* or operat*).ti,ab.
32. suburethral sling.mp.
33. abdominal sling.mp.
34. traditional sling procedure$*.tw.
35. suburethral sling procedure.tw.
36. mid$urethral sling.tw.
37. retropubic sling procedure$*.tw.
38. transobturator sling procedure$.tw.
39. TVT-Secur.mp.
40. mini-arc or mini-arc.mp.
43. solyx.mp.
44. single$incision sling$.mp.
45. mini$sling.mp.
46. Ophira.mp.
47. Tissue Fixation System.mp.
48. OR/30-47
49. ((urethra* or periurethra* or transurethra*) adj3 (agent* or bulk* or injection* or injectable*)).tw.
50. injection therapy.tw.
51. injectable$.tw.
52. (injectable$ adj2 agent$).tw.
53. (bulk$ adj3 agent$).tw.
54. autologous fat.mp.
55. Peri$urethral injection$.mp.
56. OR/38-44
57. AND/12,18,37 (this is for the sling, limit April 2018 – March 2019
58. AND/12, 18, 45 (2017-current)

CENTRAL

1. MeSH descriptor: [Urinary Incontinence, Stress] explode all trees
2. stres* near incontinen*:kw
3. stress near incontinence*:kw
4. mix* near incontinen*:kw
5. urg* near incontinen*:kw
6. stress urinary incontinence*:kw
7. occult urinary incontinence:kw
8. #1 or #2 or #3 or #4 or #5 or #6 or #7
9. MeSH descriptor: [Surgical Procedures, Operative] explode all trees
10. (surg* or surgical* or operat*):ti,ab
11. suburethral sling:kw
12. abdominal sling:kw
13. mid$urethral sling:kw
14. retropubic sling:kw
15. transobturator sling:kw
16. "mini-arc" or "mini-arc"
17. ajust
18. needleless
19. solyx
20. single$incision sling:kw
21. mini near sling:kw
22. Tissue Fixation System:kw
23. #9 or #10 or #10 or #11 or #12 or #13 or #14 or #15 or #16 or #17 or #18 or #19 or #20 or #21 or #22
24. #8 and #23
25. with Publication Year from 2018 to 2019, in Trials

injectables

1. MeSH descriptor: [Urinary Incontinence, Stress] explode all trees
2. stres* near incontinen*:kw
3. stress near incontinence*:kw
4. mix* near incontinen*:kw
5. urg* near incontinen*:kw
6. stress urinary incontinence*:kw
7. occult urinary incontinence:kw
8. #1 or #2 or #3 or #4 or #5 or #6 or #7

Repair or biceps tenotomy for SLAP lesion (13th March, 2019)

MEDLINE

1. randomized controlled trial.pt.
2. controlled clinical trial.pt.
3. randomized.ab.
7. randomly.ab.
8. trial.ti.
9. (crossover or cross-over or cross over).tw.
13. (SLAP or superior labral anterior-posterior).mp.
14. surgical procedures, operative/
15. (surg* or surgical* or operat*).ti,ab.

© 2022 Karjalainen T et al. *JAMA Network Open.*

4. randomised.ab.
5. placebo.tw.
6. clinical trials as topic.sh.
10. or/1-9
11. exp animals/ not humans.sh.
12. 10 NOT 11
16. (biceps tenodesis OR biceps tenotomy OR repair OR suture OR debridement).ti,ab.
17. OR/13-16
18. 12 and 13 and 17

EMBASE

1. Clinical Trial/
2. Randomized Controlled Trial/
3. exp randomization/
4. Single Blind Procedure/
5. Double Blind Procedure/
6. Crossover Procedure/
7. Placebo/
8. Randomi?ed controlled trial$.tw.
9. Rct.tw.
10. random allocation.tw.
11. randomly allocated.tw.
12. allocated randomly.tw.
13. (allocated adj2 random).tw.
14. Single blind$.tw.
15. Double blind$.tw.
16. ((treble or triple) adj blind$).tw.
17. placebo$.tw.
18. prospective study/
19. or/1-18
20. case study/
21. case report.tw.
22. abstract report/ or letter/
23. or/20-22
24. 19 NOT 23
25. (SLAP or superior labral anterior-posterior or superior labral anterior posterior).mp.
26. surgical procedures, operative/
27. (surg* or surgical* or operat*).ti,ab
28. (biceps tenodesis OR biceps tenotomy OR repair OR suture OR debridement).ti,ab.
29. OR/26-28
30. AND/24, 25,29

CENTRAL

1. SLAP
2. (superior labral anterior-posterior)
3. (superior labral anterior posterior)
4. #1 OR #2 OR #3

Resection of muscles for migraine (12th March, 2019)

MEDLINE

1. randomized controlled trial.pt.
2. controlled clinical trial.pt.
3. randomized.ab.
4. randomised.ab.
5. placebo.tw.
6. clinical trials as topic.sh.
7. randomly.ab.
8. trial.ti.
9. (crossover or cross-over or cross over).tw.
10. or/1-9
11. exp animals/ not humans.sh.
12. 10 NOT 11
13. Headache/ OR exp Headache Disorders/
14. exp Migraine Disorders/
15. (headach* OR migrain* OR cephalgi* OR cephalalgi*).ti,ab.
16. OR/13-15
17. surgical procedure, operative/
18. (surger* OR surgical* or operat*).tw.
19. ((nerve decompr*) OR (surgical decompr*) OR (surgical treat*)).tw.
20. OR/17-19
21. AND/12,16,20

EMBASE

1. Clinical Trial/
2. Randomized Controlled Trial/
3. exp randomization/
4. Single Blind Procedure/
12. allocated randomly.tw.
13. (allocated adj2 random).tw.
14. Single blind$.tw.
15. Double blind$.tw.
23. or/20-22
24. 19 NOT 23
25. surgical procedure/
26. (surger$ OR surgical$ or operat$).tw.

© 2022 Karjalainen T et al. *JAMA Network Open.*

5. Double Blind Procedure/
6. Crossover Procedure/
7. Placebo/
8. Randomi?ed controlled trial$.tw.
9. Rct.tw.
10. random allocation.tw.
11. randomly allocated.tw.
16. ((treble or triple) adj blind$).tw.
17. placebo$.tw.
18. prospective study/
19. or/1-18
20. case study/
21. case report.tw.
22. abstract report/ or letter/
27. OR/25-27
28. Headache/ OR exp Headache and facial pain/
29. exp Migraine/
30. (headach* OR migrain* OR cephalgi* OR cephalalgi*).ti
31. OR/29-31
32. AND/24,27,31

CENTRAL

1. MeSH descriptor Headache/ OR MeSH descriptor Headache Disorders explode all trees
2. MeSH descriptor Migraine Disorders explode all trees
3. (headach* OR migrain* OR cephalgi* OR cephalalgi*):ti,ab,kw.
4. #1 OR #2 OR #3
5. surgical procedures, operative/
6. (surg* OR surgical* or operat*):kw,ab,ti

Sphincterotomy for sphincter of oddi dysfunction (14th March, 2019)

MEDLINE

1. randomized controlled trial.pt.
2. controlled clinical trial.pt.
3. randomized.ab.
4. randomised.ab.
5. placebo.tw.
6. clinical trials as topic.sh.
7. randomly.ab.
8. trial.ti.
9. (crossover or cross-over or cross over).tw.
10. or/1-9
11. exp animals/ not humans.sh.
12. 10 NOT 11
13. sphincter of oddi.mp.
14. endoscopic sphincterotomy.mp.
15. exp surgical procedures, operative/
16. (surg* or surgical* or operat*).ti,ab.
17. or/14-16
18. and/24-25,29 (240)

EMBASE

1 Clinical Trial/ (972204)
2 Randomized Controlled Trial/ (538693)
3 exp randomization/ (81721)
4 Single Blind Procedure/ (33954)
5 Double Blind Procedure/ (160406)
6 Crossover Procedure/ (58585)
7 Placebo/ (340669)
8 Randomi?ed controlled trial$.tw. (196048)
9 Rct.tw. (31284)
10 random allocation.tw. (1931)
11 randomly allocated.tw. (31999)
12 allocated randomly.tw. (2439)
13 (allocated adj2 random).tw. (960)
14 Single blind$.tw. (22514)
15 Double blind$.tw. (200441)
16 ((treble or triple) adj blind$).tw. (954)
17 placebo$.tw. (290122)
18 prospective study/ (504017)
19 or/1-18 (2037125)
20 case study/ (68702)
21 case report.tw. (400876)
22 abstract report/ or letter/ (1086984)
23 or/20-22 (1547292)
24 19 not 23 (1986570)
25 sphincter of oddi.mp. (3272)
26 endoscopic sphincterotomy.mp. (5835)
27 exp surgical procedures, operative/ (4859006)
28 (surg* or surgical* or operat*).ti,ab. (3421933)
29 or/26-28 (6152758)
30 and/24-25,29 (240)

CENTRAL

1. sphincter of oddi
2. endoscopic sphincterotomy
3. sphincterotomy
4. surg* or surgical* or operative:kw
5. MeSH Term:[Surgical Procedures, Operative] explode all trees
6. #2 or #3 or #4 or #5
7. #1 and #6

© 2022 Karjalainen T et al. *JAMA Network Open.*

Transoral endoscopic fundoplication for gastroesophageal reflux disease (12th March, 2019)

MEDLINE

1. randomized controlled trial.pt.
2. controlled clinical trial.pt.
3. randomized.ab.
4. randomised.ab.
5. placebo.tw.
6. clinical trials as topic.sh.
7. randomly.ab.
8. trial.ti.
9. (crossover or cross-over or cross over).tw.
10. or/1-9
11. exp animals/ not humans.sh.
12. 10 NOT 11
13. transoral incisionless fundoplication.mp
14. EsophyX.mp.
15. Endocinch.mp.
16. transoral fundoplication.mp
17. endoscopic fundoplication.mp
18. OR/13-17
19. 12 and 18

EMBASE

1. Clinical Trial/
2. Randomized Controlled Trial/
3. exp randomization/
4. Single Blind Procedure/
5. Double Blind Procedure/
6. Crossover Procedure/
7. Placebo/
8. Randomi?ed controlled trial$.tw.
9. Rct.tw.
10. random allocation.tw.
11. randomly allocated.tw.
12. allocated randomly.tw.
13. (allocated adj2 random).tw.
14. Single blind$.tw.
15. Double blind$.tw.
16. ((treble or triple) adj blind$).tw.
17. placebo$.tw.
18. prospective study/
19. or/1-18
20. case study/
21. case report.tw.
22. abstract report/ or letter/
23. or/20-22
24. 19 NOT 23
25. transoral incisionless fundoplication.mp.
26. EsophyX.mp.
27. Endocinch.mp.
28. transoral fundoplication.mp.
29. endoscopic fundoplication.mp.
30. or/25-29
31. 24 and 30

CENTRAL

1. EsophyX
2. Endocinch
3. (transoral fundoplication) OR (transoral plication) OR (endoscopic plication) OR (endoscopic fundoplication)
4. TIF
5. #1 OR #2 OR #3 OR #4 OR #5

Urolift implant for prostate hyperplasia (15th March, 2019)

MEDLINE

1. Exp Prostatic Hyperplasia/
2. prostat* adj3 hyperplasia*.tw.
3. Prostate* adj3 hypertroph*.tw.
4. Prostat* adj3 adenoma*.tw.
5. BPH or BPO or BPE.tw.
6. prostat* adj3 enlarg*.tw.
7. exp prostatism/
8. prostatism.tw
11. BOO.tw.
12. OR/1-11
13. Prostatic urethral lift.tw
14. prost* ajd3 lift.tw.
15. Urolift.tw.
16. 13 or 14
17. 12 and 15
18. randomized controlled trial.pt.
21. randomized
22. placebo.tw
23. clinical trials as topic.sh
24. randomly.ab.
25. trial.ti.
26. groups.ti,ab.
27. or/17-25
28. animals not (humans and animals).sh

© 2022 Karjalainen T et al. *JAMA Network Open.*

9. exp Urinary Bladder Neck Obstruction/
10. Bladder* adj3 obstruct*.tw.
19. controlled clinical trial.pt.
20. randomized
29. 26 not 27
30. 16 and 28

EMBASE

1. Clinical Trial/
2. Randomized Controlled Trial/
3. exp randomization/
4. Single Blind Procedure/
5. Double Blind Procedure/
6. Crossover Procedure/
7. Placebo/
8. Randomi?ed controlled trial$.tw.
9. Rct.tw.
10. random allocation.tw.
11. randomly allocated.tw.
12. allocated randomly.tw.
13. (allocated adj2 random).tw.
14. Single blind$.tw.
15. Double blind$.tw.
16. ((treble or triple) adj blind$).tw.
17. placebo$.tw.
18. prospective study/
19. or/1-18
20. case study/
21. case report.tw.
22. abstract report/ or letter/
23. or/20-22
24. 19 NOT 23
25. Prostatic urethral lift.tw
26. prost$ lift.tw.
27. Urolift.tw.
28. OR/25-27
29. Exp Prostatic Hyperplasia/
30. prostat$ adj3 hyperplasia$.tw.
31. Prostate$ adj3 hypertroph$.tw.
32. Prostat$ adj3 adenoma$.tw.
33. BPH or BPO or BPE.tw.
34. prostat$ adj3 enlarg$.tw.
35. exp prostatism/
36. prostatism.tw.
37. exp Urinary Bladder Neck Obstruction/
38. Bladder$ adj3 obstruct$.tw.
39. BOO.tw.
40. OR/29-39
41. and/24,28,40

CENTRAL

Urolift

Prostatic urethral lift

prost* lift:kw

Vertebroplasty for vertebral compression fracture (14th March, 2019)

Updated search from Buchbinder R, Johnston RV, Rischin KJ,et al. Percutaneous vertebroplasty for osteoporotic vertebral compression fracture. *Cochrane Database Syst Rev* 2018;4:CD006349. doi: 10.1002/14651858.CD006349.pub3.

MEDLINE

1. randomized controlled trial.pt.
2. controlled clinical trial.pt.
3. randomized.ab.
4. randomised.ab.
5. placebo.tw.
6. clinical trials as topic.sh.
7. randomly.ab.
8. trial.ti.
9. (crossover or cross-over or cross over).tw.
10. or/1-9
11. exp animals/ not humans.sh.
12. 10 NOT 11
13. exp Spine/
14. (spine or spinal or vertebra$).tw.
15. exp Fractures, Bone/
16. fractur$.ti.
17. 13 or 14
18. 16 or 16
19. 17 and 18
20. exp Spinal Fractures/
21. 19 or 20
22. exp Bone Cements/
23. exp Methylmethacrylates/
24. methacrylate$.tw.
25. bone cement$.tw.
26. exp Fracture Fixation, Internal/
27. exp Vertebroplasty/
28. vertebroplast$.tw.
29. cementoplast$.tw.
30. sacroplast$.tw. (114)
31. or/22-30
32. and/12,21,31
33. 2017-current

EMBASE

© 2022 Karjalainen T et al. *JAMA Network Open.*

1. Clinical Trial/
2. Randomized Controlled Trial/
3. exp randomization/
4. Single Blind Procedure/
5. Double Blind Procedure/
6. Crossover Procedure/
7. Placebo/
8. Randomi?ed controlled trial$.tw.
9. Rct.tw.
10. random allocation.tw.
11. randomly allocated.tw.
12. allocated randomly.tw.
13. (allocated adj2 random).tw.
14. Single blind$.tw.
15. Double blind$.tw.
16. ((treble or triple) adj blind$).tw.
17. placebo$.tw.
18. prospective study/
19. or/1-18
20. case study/
21. case report.tw.
22. abstract report/ or letter/
23. or/20-22
24. 19 NOT 23
19. exp Spine/
20. (spine or spinal or vertebra$).tw.
21. exp Fractures, Bone/
22. fractur$.ti.
23. 33 or 34
24. 35 or 36
25. 37 and 38
26. exp Spinal Fractures/
27. 39 or 40
28. exp Bone Cements/
29. exp Methylmethacrylates/
30. methacrylate$.tw.
31. bone cement$.tw.
32. exp Fracture Fixation, Internal/
33. exp Vertebroplasty/
34. vertebroplast$.tw.
35. cementoplast$.tw.
36. sacroplast$.tw. (114)
37. or/42-50
38. and/12,41,51
    2017-current

CENTRAL

1 exp Spine/ (4032)
2 (spine or spinal or vertebra$).tw. (20306)
3 exp Fractures, Bone/ (3949)
4 fractur$.ti. (6791)
5 1 or 2 (21641)
6 3 or 4 (8007)
7 5 and 6 (1504)
8 exp Spinal Fractures/ (561)
9 7 or 8 (1528)
10 exp Bone Cements/ (769)
11 exp Methylmethacrylates/ (389)
12 methacrylate$.tw. (251)
13 bone cement$.tw. (201)
14 exp Fracture Fixation, Internal/ (1077)
15 exp Vertebroplasty/ (112)
16 vertebroplast$.tw. (202)
17 cementoplast$.tw. (10)
18 sacroplast$.tw. (2)
19 or/10-18 (2421)
20 9 and 19 (244)

© 2022 Karjalainen T et al. *JAMA Network Open.*

eAppendix 2. List of Included Trials

Condition/Interventions that have both Placebo controls and trials with non-operative controls were included in the meta-regression unless otherwise indicated (groups from studies with both placebo and non-operative controls were included in both sides halving the n in the active group when in the same meta-analysis)

* = Trial met the inclusion criteria but did not provide sufficient data to be included in the analyses

Low-flow shunt for Alzheimer's disease

Placebo controls

1. Silverberg GD, Mayo M, Saul T, et al. Continuous CSF drainage in AD: Results of a double-blind, randomized, Placebo controls study. *Neurology.* 2008;71(3):202–9. doi:10.1212/01.wnl.0000316197.04157.6f.

Non-operative contols

2. Silverberg GD, Levinthal E, Sullivan EV, et al. Assessment of low-flow CSF drainage as a treatment for AD: Results of a randomized pilot study. *Neurology.* 2002;59(8):1139–45. doi:10.1212/01.WNL.0000031794.42077.A1.

Brain stimulation for cervical dystonia

Placebo controls

3. Kupsch A, Benecke R, Müller J, et al. Pallidal deep-brain stimulation in primary generalized or segmental dystonia. *N Engl J Med.* 2006;355(19):1978–90. doi:10.1056/nejmoa063618.
4. Volkmann J, Mueller J, Deuschl G, et al. Pallidal neurostimulation in patients with medication-refractory cervical dystonia: a randomised, sham-controlled trial. *Lancet Neurol*. 2014;13(9):875-884. doi:10.1016/S1474-4422(14)70143-7

Adhesiolysis for chronic abdominal pain

Placebo controls

5. Cheong YC, Reading I, Bailey S, et al. Should women with chronic pelvic pain have adhesiolysis? *BMC Womens Health.* Mar 2014;14:36. doi:10.1186/1472-6874-14-36.
6. Swank DJ, Swank-Bordewijk SCG, Hop WCJ, et al. Laparoscopic adhesiolysis in patients with chronic abdominal pain: A blinded randomised controlled multi-centre trial. *Lancet.* 2003;361(9365):1247–51. doi:10.1016/S0140-6736(03)12979-0.

Non-operative contols

7. Peters AA, Trimbos-Kemper GC, Admiraal C, et al. A randomized clinical trial on the benefit of adhesiolysis in patients with intraperitoneal adhesions and chronic pelvic pain. *Br J Obstet Gynaecol.* 1992;99(1):59–62. doi:10.1111/j.1471-0528.1992.tb14394.x.

Laparoscopic uterosacral nerve ablation for chronic abdominal or pelvic pain

Placebo controls

8. Lichten EM, Bombard J. Surgical treatment of primary dysmenorrhea with laparoscopic uterine nerve ablation. *J Reprod Med.* 1987;32(1):37–41.
9. Daniels J, Gray R, Hills RK, et al. Laparoscopic uterosacral nerve ablation for alleviating chronic pelvic pain: A randomized controlled trial. *JAMA.* 2009;302(9):955–61. doi:10.1001/jama.2009.1268.
10. El-Din Shawki H. The efficacy of laparoscopic uterosacral nerve ablation (LUNA) in the treatment of unexplained chronic pelvic pain: A randomized controlled trial. *Gynecol Surg.* 2011;8(1):31–9. doi:10.1007/s10397-010-0612-1.
11. Johnson NP, Farquhar CM, Crossley S, et al. A double-blind randomised controlled trial of laparoscopic uterine nerve ablation for women with chronic pelvic pain. *BJOG.* 2004;111(9):950–9. doi:10.1111/j.1471-0528.2004.00233.x.

© 2022 Karjalainen T et al. *JAMA Network Open.*

12. *Sutton C, Pooley AS, Jones KD, Dover RW, Haines P. A prospective, randomized, double-blind controlled trial of laparoscopic uterine nerve ablation in the treatment of pelvic pain associated with endometriosis. *Gynaecological Endoscopy.* 2001;10(4):217–22. doi.org/10.1046/j.1365-2508.2001.00451.x.

Excision or ablation for endometriosis

Placebo controls

13. Abbott J, Hawe J, Hunter D, Holmes M, Finn P, Garry R. Laparoscopic excision of endometriosis: A randomized, Placebo controls trial. *Fertil Steril.* 2004;82(4):878–84. doi:10.1016/j.fertnstert.2004.03.046.
14. Jarrell J, Mohindra R, Ross S, Taenzer P, Brant R. Laparoscopy and reported pain among patients with endometriosis. *J Obstet Gynaecol Can.* 2005;27(5):477–85. doi:10.1016/S1701-2163(16)30531-X.
15. *Tutunaru D, Vladareanu R, Dumitrascu MC, Alexandru B. Placebo effect of diagnostic laparoscopy alone in mild endometriosis. *J Gynaecol Obstet.* 2006;2:144.
16. Sutton CJG, Ewen SP, Whitelaw N, Haines P. Prospective, randomized, double-blind, controlled trial of laser laparoscopy in the treatment of pelvic pain associated with minimal, mild, and moderate endometriosis. *Fertil Steril.* 1994;62(4):696–700. doi:10.1016/s0015-0282(16)56990-8.

Non-operative contols

17. Alkatout I, Mettler L, Beteta C, et al. Combined surgical and hormone therapy for endometriosis is the most effective treatment: prospective, randomized, controlled trial. *J Minim Invasive Gynecol.* 2013;20(4):473–81. doi:10.1016/j.jmig.2013.01.019.

Left internal mammary artery ligation for coronary heart disease

Placebo controls

18. Cobb LA, Thomas GI, Dillard DH, Merendino KA, Bruce RA. An evaluation of internal-mammary-artery ligation by a double-blind technic. *N Engl J Med.* 1959;260(22):1115–8. doi:10.1056/nejm195905282602204.
19. Dimond EG, Kittle CF, Crockett JE. Comparison of internal mammary artery ligation and sham operation for angina pectoris*. *Am J Cardiol.* Apr 1960;5:483–6. doi:10.1016/0002-9149(60)90105-3.

Transoral endoscopic fundoplication for gastroesophageal reflux disease

Placebo and non-operative contols

20. Schwartz MP, Wellink H, Gooszen HG, Conchillo JM, Samsom M, Smout AJPM. Endoscopic gastroplication for the treatment of gastro-oesophageal reflux disease: A randomised, sham-controlled trial. *Gut.* 2007;56(1):20–8. doi:10.1136/gut.2006.096842.

Placebo controls

21. Hunter JG, Kahrilas PJ, Bell RCW, et al. Efficacy of transoral fundoplication vs omeprazole for treatment of regurgitation in a randomized controlled trial. *Gastroenterology.* 2015;148(2):324-333.e5. doi:10.1053/j.gastro.2014.10.009.
22. Håkansson B, Montgomery M, Cadiere GB, et al. Randomised clinical trial: transoral incisionless fundoplication vs. sham intervention to control chronic GERD. *Aliment Pharmacol Ther.* 2015;42(11-12):1261–70. doi:10.1111/apt.13427.
23. Kalapala R, Karyampudi A, Nabi Z, Chavan R, Santosh D, Duvvur NR. Endoscopic full thickness plication (gerd-x) for the treatment of ppi dependent gerd: a randomized, single blinded, sham controlled trial. *Gastrointest Endosc.* 2018;87(6):AB263–4. doi:10.1016/j.gie.2018.04.1566.
24. Montgomery M, Håkanson B, Ljungqvist O, Ahlman B, Thorell A. Twelve months' follow-up after treatment with the EndoCinch endoscopic technique for gastro-oesophageal reflux disease: A randomized, Placebo controls study. *Scand J Gastroenterol.* 2006;41(12):1382–9. doi:10.1080/00365520600735738.
25. Rothstein R, Filipi C, Caca K, et al. Endoscopic full-thickness plication for the treatment of gastroesophageal reflux disease: A randomized, sham-controlled trial. *Gastroenterology.* 2006;131(3):704–12. doi:10.1053/j.gastro.2006.07.004.

Non-operative contols

26. Trad KS, Barnes WE, Simoni G, et al. Transoral incisionless fundoplication effective in eliminating GERD symptoms in partial responders to proton pump inhibitor therapy at 6 months: The TEMPO randomized clinical trial. *Surg Innov.* 2015;22(1):26–40. doi:10.1177/1553350614526788.
27. Witteman BPL, Conchillo JM, Rinsma NF, et al. Randomized controlled trial of transoral incisionless fundoplication vs. proton pump inhibitors for treatment of

© 2022 Karjalainen T et al. *JAMA Network Open.*

gastroesophageal reflux disease. *Am J Gastroenterol.* 2015;110(4):531–42. doi:10.1038/ajg.2015.28.
Ablation for infertility with endometriosis
Placebo controls
28. Moini A, Bahar L, Ashrafinia M, Eslami B, Hosseini R, Ashrafinia N. Fertility outcome after operative laparoscopy versus no treatment in infertile women with minimal or mild endometriosis. *Int J Fertil Steril.* 2012;5(4):235-40.
Non-operative contols
Alkatout I, Mettler L, Beteta C, et al. Combined surgical and hormone therapy for endometriosis is the most effective treatment: prospective, randomized, controlled trial. *J Minim Invasive Gynecol.* 2013;20(4):473–81. doi:10.1016/j.jmig.2013.01.019. (also included in **Excision or ablation for endometriosis)**
29. Marcoux S, Maheux R, Bérubé S. Laparoscopic surgery in infertile women with minimal or mild endometriosis. *N Engl J Med.* 1997;337(4):217–22. doi:10.1056/nejm199707243370401.
30. Gad MS, Badroui MHH. Evidence-based therapy for infertility associated with early stage endometriosis. *J Gynaecol Endometr.* 2012;531–687:S548.
31. Parazzini F, Cintio E Di, Chatenoud L, et al. Ablation of lesions or no treatment in minimal-mild endometriosis in infertile women: A randomized trial. *Hum Reprod.* 1999;14(5):1332–4. doi:10.1093/humrep/14.5.1332.
Arthroscopic partial meniscectomy for knee meniscal tear
Placebo controls
32. Sihvonen R, Paavola M, Malmivaara A, et al. Arthroscopic partial meniscectomy versus sham surgery for a degenerative meniscal tear. *N Engl J Med.* 2013;369(26):2515–24. doi:10.1056/NEJMoa1305189.
33. Roos EM, Hare KB, Nielsen SM, Christensen R, Lohmander LS. Better outcome from arthroscopic partial meniscectomy than skin incisions only? A sham-controlled randomised trial in patients aged 35-55 years with knee pain and an MRI-verified meniscal tear. *BMJ Open.* 2018;8(2):e019461. doi:10.1136/bmjopen-2017-019461.
Non-Placebo controls
34. Gauffin H, Tagesson S, Meunier A, Magnusson H, Kvist J. Knee arthroscopic surgery is beneficial to middle-aged patients with meniscal symptoms: a prospective, randomised, single-blinded study. *Osteoarthritis Cartilage.* 2014;22(11):1808–16. doi:10.1016/j.joca.2014.07.017.
35. Herrlin S, Hållander M, Wange P, Weidenhielm L, Werner S. Arthroscopic or conservative treatment of degenerative medial meniscal tears: a prospective randomised trial. *Knee Surg Sports Traumatol Arthrosc.* 2007;15(4):393–401. doi:10.1007/s00167-006-0243-2.
36. Katz JN, Brophy RH, Chaisson CE, et al. Surgery versus physical therapy for a meniscal tear and osteoarthritis. *N Engl J Med.* 2013;368(18):1675–84. doi:10.1056/NEJMoa1301408.
37. Østerås H, Østerås B, Torstensen TA. Medical exercise therapy, and not arthroscopic surgery, resulted in decreased depression and anxiety in patients with degenerative meniscus injury. *J Bodyw Mov Ther.* 2012;16(4):456–63. doi:10.1016/j.jbmt.2012.04.003.
38. Stensrud S, Risberg MA, Roos EM. Effect of exercise therapy compared with arthroscopic surgery on knee muscle strength and functional performance in middle-aged patients with degenerative meniscus tears: a 3-mo follow-up of a randomized controlled trial. *Am J Phys Med Rehabil.* 2015;94(6):460–73. doi:10.1097/PHM.0000000000000209.
39. van de Graaf VA, Noorduyn JCA, Willigenburg NW, et al. Effect of early surgery vs physical therapy on knee function among patients with nonobstructive meniscal tears: the ESCAPE randomized clinical trial. *JAMA.* 2018;320(13):1328–37 doi:10.1001/jama.2018.13308.
40. Yim JH, Seon JK, Song EK, et al. A comparative study of meniscectomy and nonoperative treatment for degenerative horizontal tears of the medial meniscus. *Am J Sports Med.* 2013;41(7):1565–70. doi:10.1177/0363546513488518.
Arthroscopic debridement for knee osteoarthritis
Placebo controls

© 2022 Karjalainen T et al. *JAMA Network Open.*

41. Moseley JB, O'Malley K, Petersen NJ, et al. A controlled trial of arthroscopic surgery for osteoarthritis of the knee. *N Engl J Med.* 2002;347(2):81–8. doi:10.1056/NEJMoa013259.

Non-operative contols

42. Kirkley A, Birmingham TB, Litchfield RB, et al. A randomized trial of arthroscopic surgery for osteoarthritis of the knee. *N Engl J Med.* 2008;359(11):1097–107. doi:10.1056/nejmoa0708333.
43. Merchan ECR, Galindo E. Arthroscope-guided surgery versus nonoperative treatment for limited degenerative osteoarthritis of the femorotibial joint in patients over 50 years of age: A prospective comparative study. *Arthroscopy.* 1993;9(6):663–7. doi:10.1016/S0749-8063(05)80503-1.

Endolymphatic shunt for Meniere's disease

Placebo controls

44. Thomsen J, Bretlau P, Tos M, Johnsen NJ. Placebo effect in surgery for Ménière's disease. A double-blind, Placebo controls study on endolymphatic sac shunt surgery. *Arch Otolaryngol.* 1981;107(5):271–7. doi:10.1001/archotol.1981.00790410009002.

Resection of muscles for migraine

Placebo controls

45. Guyuron B, Reed D, Kriegler JS, Davis J, Pashmini N, Amini S. A Placebo controls surgical trial of the treatment of migraine headaches. *Plast Reconstr Surg.* 2009;124(2):461–8. doi:10.1097/PRS.0b013e3181adcf6a.

Non-operative contols

46. Guyuron B, Kriegler JS, Davis J, Amini SB. Comprehensive surgical treatment of migraine headaches. *Plast Reconstr Surg*. 2005;115(1):1-9.

Endoscopic gastric plication for obesity

Placebo controls

47. Eid GM, McCloskey CA, Eagleton JK, Lee LB, Courcoulas AP. StomaphyX vs a sham procedure for revisional surgery to reduce regained weight in roux-en-y gastric bypass patients a randomized clinical trial. *JAMA Surg.* 2014;149(4):372–9. doi:10.1001/jamasurg.2013.4051.
48. Sullivan S, Swain JM, Woodman G, et al. Randomized sham-controlled trial evaluating efficacy and safety of endoscopic gastric plication for primary obesity: The ESSENTIAL trial. *Obesity (Silver Spring).* 2017;25(2):294–301. doi:10.1002/oby.21702.

Non-Placebo controls

49. Miller K, Turró R, Greve JW, Bakker CM, Buchwald JN, Espinos JC. MILEPOST multicenter randomized controlled trial: 12-month weight loss and satiety outcomes after pose [SM] vs. medical therapy. *Obes Surg.* 2017;27(2):310–22. doi:10.1007/s11695-016-2295-9.

Vagal nerve stimulation for obesity

Placebo controls

50. Ikramuddin S, Blackstone RP, Brancatisano A, et al. Effect of reversible intermittent intra-abdominal vagal nerve blockade on morbid obesity: The ReCharge randomized clinical trial. *JAMA.* 2014;312(9):915-22. doi:10.1001/jama.2014.10540 [published Online First: 2014].
51. Sarr MG, Billington CJ, Brancatisano R, et al. The EMPOWER Study: Randomized, prospective, double-blind, multicenter trial of vagal blockade to induce weight loss in morbid obesity. *Obes Surg.* 2012;22(11):1771–82. doi:10.1007/s11695-012-0751-8.

Transoral outlet suture for obesity

Placebo controls

52. Thompson CC, Chand B, Chen YK, et al. Endoscopic suturing for transoral outlet reduction increases weight loss after Roux-en-Y gastric bypass surgery. *Gastroenterology.* 2013;145(1):129-137.e3. doi: 10.1053/j.gastro.2013.04.002.

Prophylactic midurethral sling for occult urinary incontinence

Placebo controls

53. Wei JT, Nygaard I, Richter HE, et al. A midurethral sling to reduce incontinence after vaginal prolapse repair. *N Engl J Med.* 2012;366(25):2358–67. doi:10.1056/nejmoa1111967.
Non-operative contols
54. Fuentes AE. A prospective randomized controlled trial comparing vaginal prolapse repair with and without tension free vaginal tape transobturator taper (TVTO) in women with severe genital prolapse and occult stress incontinence: long term follow up. *Int Urogynecol J.* 2011;22(Suppl 1):S60-61. https://doi.org/10.1007/s00192-011-1434-z
55. Schierlitz L, Dwyer PL, Rosamilia A, et al. Pelvic organ prolapse surgery with and without tension-free vaginal tape in women with occult or asymptomatic urodynamic stress incontinence: A randomised controlled trial. *Int Urogynecol J.* 2014;25(1):33–40. doi:10.1007/s00192-013-2150-7.
56. van der Ploeg JM, Oude Rengerink K, van der Steen A, Schagen van Leeuwen JH, Huub van der Vaart CH, Roovers JPWR. Vaginal prolapse repair with or without a midurethral sling in women with genital prolapse and occult stress urinary incontinence: a randomized trial. *Int Urogynecol J.* 2016;27(7):1029–38. doi:10.1007/s00192-015-2924-1.
Transplantation AAV2 neurturin gene for Parkinson's disease
Placebo controls
57. LeWitt PA, Rezai AR, Leehey MA, et al. AAV2-GAD gene therapy for advanced Parkinson's disease: A double-blind, sham-surgery controlled, randomised trial. *Lancet Neurol.* 2011;10(4):309–19. doi:10.1016/S1474-4422(11)70039-4.
58. Olanow C, Bartus RT, Baumann TL, et al. Gene delivery of neurturin to putamen and substantia nigra in Parkinson disease: A double-blind, randomized, controlled trial. *Ann Neurol.* 2015;78(2):248-57. doi:10.1002/ana.24436.
59. Marks WJ, Bartus RT, Siffert J, et al. Gene delivery of AAV2-neurturin for Parkinson's disease: A double-blind, randomised, controlled trial. *Lancet Neurol.* 2010;9(12):1164–72. doi:10.1016/S1474-4422(10)70254-4.
Transplantation of human retinal pigment epithelial cells for Parkinson's disease
Placebo controls
60. Gross RE, Watts RL, Hauser RA, et al. Intrastriatal transplantation of microcarrier-bound human retinal pigment epithelial cells versus sham surgery in patients with advanced Parkinson's disease: A double-blind, randomised, controlled trial. *Lancet Neurol.* 2011;10(6):509-19. doi:10.1016/S1474-4422(11)70097-7.
Transplantation of fetal cells for Parkinson's disease
Placebo controls
61. Olanow CW, Goetz CG, Kordower JH, et al. A double-blind controlled trial of bilateral fetal nigral transplantation in Parkinson's disease. *Ann Neurol.* 2003;54(3):403–14. doi:10.1002/ana.10720.
62. Freed CR, Greene PE, Breeze RE, et al. Transplantation of embryonic dopamine neurons for severe Parkinson's disease. *N Engl J Med.* 2001;344(10):710–9. doi:10.1056/nejm200103083441002.
Non-operative contols
63. Spencer DD, Robbins RJ, Naftolin F, et al. Unilateral transplantation of human fetal mesencephalic tissue into the caudate nucleus of patients with Parkinson's disease. *N Engl J Med.* 1992;327(22):1541–8. doi:10.1056/nejm199211263272201.
Scalpel debridement for plantar callus
Placebo controls
64. *Aragua Garcia C, Corbi Soler F. Effect of debridement of plantar hyperkeratoses on gait in older people – An exploratory trial. *Arch Gerontol Geriatr.* Sep-Oct 2018;78:7–13. doi:10.1016/j.archger.2018.05.017.
65. Davys HJ, Turner DE, Helliwell PS, Conaghan PG, Emery P, Woodburn J. Debridement of plantar callosities in rheumatoid arthritis: A randomized controlled trial. *Rheumatology (Oxford).* 2005;44(2):207-10. doi:10.1093/rheumatology/keh435.

© 2022 Karjalainen T et al. *JAMA Network Open.*

66. Landorf KB, Morrow A, Spink MJ, et al. Effectiveness of scalpel debridement for painful plantar calluses in older people: A randomized trial. *Trials.* Aug 2013;14:243. doi:10.1186/1745-6215-14-243.

Non-operative contols

67. Siddle HJ, Redmond AC, Waxman R, et al. Debridement of painful forefoot plantar callosities in rheumatoid arthritis: The CARROT randomised controlled trial. *Clin Rheumatol.* 2013;32(5):567-74. doi:10.1007/s10067-012-2134-x.

Urolift® implant for prostate hyperplasia

Placebo controls

68. Roehrborn CG, Gange SN, Shore ND, et al. The prostatic urethral lift for the treatment of lower urinary tract symptoms associated with prostate enlargement due to benign prostatic hyperplasia: The L.I.F.T. study. *J Urol.* 2013;190(6):2161–7. doi:10.1016/j.juro.2013.05.116.

Arthroscopic subacromial decompression for rotator cuff disease

Placebo and Non-operative contols

69. Beard DJ, Rees JL, Cook JA, et al. Arthroscopic subacromial decompression for subacromial shoulder pain (CSAW): a multicentre, pragmatic, parallel group, Placebo controls, three-group, randomised surgical trial. *Lancet.* 2018;391(10118):329–38. doi:10.1016/S0140-6736(17)32457-1.
70. Paavola M, Malmivaara A, Taimela S, et al. Subacromial decompression versus diagnostic arthroscopy for shoulder impingement: randomised, placebo surgery controlled clinical trial. *BMJ.* Jul 2018;362:k2860. doi:10.1136/bmj.k2860.

Non-operative contols

71. Brox JI, Staff PH, Ljunggren AE, Brevik JI. Arthroscopic surgery compared with supervised exercises in patients with rotator cuff disease (stage II impingement syndrome). *BMJ.* 1993;307(6909):899–903. doi:10.1136/bmj.307.6909.899.
72. Farfaras S, Sernert N, Hallström E, Kartus J. Comparison of open acromioplasty, arthroscopic acromioplasty and physiotherapy in patients with subacromial impingement syndrome: a prospective randomised study. *Knee Surg Sports Traumatol Arthrosc.* 2016;24(7):2181–91. doi:10.1007/s00167-014-3416-4.
73. Haahr JP, Østergaard S, Dalsgaard J, et al. Exercises versus arthroscopic decompression in patients with subacromial impingement: A randomised, controlled study in 90 cases with a one year follow up. *Ann Rheum Dis.* 2005;64(5):760–4. doi:10.1136/ard.2004.021188.
74. Ketola S, Lehtinen J, Arnala I, et al. Does arthroscopic acromioplasty provide any additional value in the treatment of shoulder impingement syndrome? A two-year randomised controlled trial. *J Bone Joint Surg Br.* 2009;91(10):1326–34. doi:10.1302/0301-620X.91B10.22094.
75. Peters G, Kohn D. Mittelfristige klinische Resultate nach operativer versus konservativer Behandlung des subakromialen Impingementsyndroms. *Unfallchirurg.* Aug 1997;100:623-29. doi:10.1007/s001130050167.
76. Rahme H, Solem-Bertoft E, Westerberg CE, Lundberg E, Sörensen S, Hilding S. . The subacromial impingement syndrome. A study of results of treatment with special emphasis on predictive factors and pain-generating mechanisms. *Scand J Rehabil Med.* 1998;30(4):253–62. doi:10.1080/003655098444002.

Repair or biceps tenotomy for SLAP lesion

Placebo controls

77. Schrøder CP, Skare Ø, Reikerås O, Mowinckel P, Brox JI. Sham surgery versus labral repair or biceps tenodesis for type II SLAP lesions of the shoulder: A three-armed randomised clinical trial. *Br J Sports Med.* 2017;51(24):1759–66. doi:10.1136/bjsports-2016-097098.

Palatal implants for sleep apnea

Placebo controls

78. Friedman M, Schalch P, Lin HC, Kakodkar KA, Joseph NJ, Mazloom N. Palatal implants for the treatment of snoring and obstructive sleep apnea/hypopnea syndrome. *Otolaryngol Head Neck Surg.* 2008;138(2):209-16. doi:10.1016/j.otohns.2007.10.026.
79. Gillespie MB, Wylie PE, Lee-Chiong T, Rapoport DM. Effect of palatal implants on continuous positive airway pressure and compliance. *Otolaryngol Head Neck Surg.* 2011;144(2):230-6. doi:10.1177/0194599810392173.

© 2022 Karjalainen T et al. *JAMA Network Open.*

80. Maurer JT, Sommer JU, Hein G, Hörmann K, Heiser C, Stuck BA. Palatal implants in the treatment of obstructive sleep apnea: A randomised, Placebo controls single-centre trial. *Eur Arch Otorhinolaryngol.* 2012;269(7):1851-6. doi:10.1007/s00405-011-1920-4.
81. Steward DL, Huntley TC, Woodson BT, Surdulescu V. Palate implants for obstructive sleep apnea: Multi-institution, randomized, Placebo controls study. *Otolaryngol Head Neck Surg.* 2008;139(4):506-10. doi:10.1016/j.otohns.2008.07.021.

Nasal surgery for sleep apnea

Placebo controls

82. Koutsourelakis I, Georgoulopoulos G, Perraki E, Vagiakis E, Roussos C, Zakynthinos SG. Randomised trial of nasal surgery for fixed nasal obstruction in obstructive sleep apnoea. *Eur Respir J.* 2008;31(1):110-7. doi:10.1183/09031936.00087607.

Sphincterotomy for sphincter-of-oddi dysfunction

Placebo controls

83. Cotton PB, Durkalski V, Romagnuolo J, et al. Effect of endoscopic sphincterotomy for suspected sphincter of oddi dysfunction on pain-related disability following cholecystectomy: The EPISOD randomized clinical trial. *JAMA.* 2014;311(20):2101-9. doi:10.1001/jama.2014.5220.
84. Geenen JE, Hogan WJ, Dodds WJ, Toouli J, Venu RP. The efficacy of endoscopic sphincterotomy after cholecystectomy in patients with sphincter-of-Oddi dysfunction. *N Engl J Med.* 1989;320(2):82-7. doi:10.1056/nejm198901123200203.
85. Sherman S, Lehman G, P J. Efficacy of endoscopic sphincterotomy and surgical sphincteroplasty for patients with sphincter of Oddi dysfunction (SOD); randomized controlled study. *Gastrointest Endosc.* 1994;140:125A.
86. Toouli J, Roberts-Thomson IC, Kellow J, et al. Manometry based randomised trial of endoscopic sphincterotomy for sphincter of Oddi dysfunction. *Gut.* 2000;46(1):98-102. doi:10.1136/gut.46.1.98.

Autologous olfactory transplant for spinal cord injury

Placebo controls

87. Chen L, Huang H, Xi H, et al. A prospective randomized double-blind clinical trial using a combination of olfactory ensheathing cells and Schwann cells for the treatment of chronic complete spinal cord injuries. *Cell Transplant.* 2014;23(Suppl 1):S35-44. doi:10.3727/096368914x685014.

Non-operative contols

88. Wang S, Lu J, Li YA, et al. Autologous olfactory lamina propria transplantation for chronic spinal cord injury: Three-year follow-up outcomes from a prospective double-blinded clinical trial. *Cell Transplant.* 2016;25(1):141-57. doi:10.3727/096368915X688065.

Deep brain stimulation for tardive dystonia

Placebo controls

89. Gruber D, Südmeyer M, Deuschl G, et al. Neurostimulation in tardive dystonia/dyskinesia: A delayed start, sham stimulation-controlled randomized trial. *Brain Stimul.* 2018;11(6):1368–77. doi:10.1016/j.brs.2018.08.006.

ECRB release for tennis elbow

Placebo controls

90. Kroslak M, Murrell GAC. Surgical treatment of lateral epicondylitis: a prospective, randomized, double-blinded, Placebo controls clinical trial. *Am J Sports Med.* 2018;46(5):1106–13. doi:10.1177/0363546517753385.

Autologous fat transplant for stress urinary incontinence

91. Lee PE, Kung RC, Drutz HP. Periurethral autologous fat injection as treatment for female stress urinary incontinence: A randomized double-blind controlled trial. *J Urol.* 2001;165(1):153-8. doi:10.1097/00005392-200101000-00037.

Vertebroplasty for vertebral compression fracture

Placebo controls

92. Buchbinder R, Osborne RH, Ebeling PR, et al. A randomized trial of vertebroplasty for painful osteoporotic vertebral fractures. *N Engl J Med.* Aug 2009;361:557-

© 2022 Karjalainen T et al. *JAMA Network Open.*

68. doi:10.1056/NEJMoa0900429.
93. Clark W, Bird P, Gonski P, et al. Safety and efficacy of vertebroplasty for acute painful osteoporotic fractures (VAPOUR): a multicentre, randomised, double-blind, Placebo controls trial. *Lancet.* 2016;388(10052):1408-1416. doi:10.1016/S0140-6736(16)31341-1.
94. Firanescu CE, de Vries J, Lodder P, et al. Vertebroplasty versus sham procedure for painful acute osteoporotic vertebral compression fractures (VERTOS IV): Randomised sham controlled clinical trial. *BMJ.* May 2018;361:k1551. doi:10.1136/bmj.k1551.
95. Hansen EJ, Simony A, Rousing R, et al. Double blind Placebo controls trial of percutaneous vertebroplasty (VOPE). *Glob Spine J.* 2016;6(1_suppl):s-0036-1582763-s-0036-1582763. https://doi.org/10.1055/s-0036-1582763 .
96. Kallmes DF, Comstock BA, Heagerty PJ, et al. A randomized trial of vertebroplasty for osteoporotic spinal fractures. *N Engl J Med.* Aug 2009;361:569–79. doi:10.1056/NEJMoa0900563.

Non-operative contols

97. Blasco J, Martinez-Ferrer A, Macho J, et al. Effect of vertebroplasty on pain relief, quality of life, and the incidence of new vertebral fractures: A 12-month randomized follow-up, controlled trial. *J Bone Miner Res.* 2012;27(5):1159–66. doi:10.1002/jbmr.1564.
98. Chen D, An ZQ, Song S, Tang JF, Qin H. Percutaneous vertebroplasty compared with conservative treatment in patients with chronic painful osteoporotic spinal fractures. *J Clin Neurosci.* 2014;21(3):473-7. doi:10.1016/j.jocn.2013.05.017
99. Farrokhi MR, Alibai E, Maghami Z. Randomized controlled trial of percutaneous vertebroplasty versus optimal medical management for the relief of pain and disability in acute osteoporotic vertebral compression fractures. *J Neurosurg Spine.* 2011;14(5):561-9. doi:10.3171/2010.12.SPINE10286.
100. Klazen CAH, Lohle PNM, de Vries J, et al. Vertebroplasty versus conservative treatment in acute osteoporotic vertebral compression fractures (Vertos II): an open-label randomised trial. *Lancet.* 2010;376(9746):1085–92. doi:10.1016/S0140-6736(10)60954-3.
101. Rousing R, Hansen KL, Andersen MO, Jespersen SM, Thomsen K, Lauritsen JM. Twelve-months follow-up in forty-nine patients with acute/semiacute osteoporotic vertebral fractures treated conservatively or with percutaneous vertebroplasty: A clinical randomized study. *Spine (Phila Pa 1976).* 2010;35(5):478-82. doi:10.1097/BRS.0b013e3181b71bd1.
102. Voormolen MHJ, Lohle PN, Lampmann LE, et al. Prospective clinical follow-up after percutaneous vertebroplasty in patients with painful osteoporotic vertebral compression fractures. *J Vasc Interv Radiol.* 2006;17(8):1313–20. doi:10.1097/01.RVI.0000231952.75209.4A.
103. Yang EZ, Xu JG, Huang GZ, et al. Percutaneous vertebroplasty versus conservative treatment in aged patients with acute osteoporotic vertebral compression fractures. *Spine (Phila Pa 1976).* 2016;41(8):653–60. doi:10.1097/BRS.0000000000001298.

© 2022 Karjalainen T et al. *JAMA Network Open.*
